# Long G4-rich enhancers target promoters via a G4 DNA-based mechanism

**DOI:** 10.1093/nar/gkae1180

**Published:** 2024-12-10

**Authors:** Jeffrey D DeMeis, Justin T Roberts, Haley A Delcher, Noel L Godang, Alexander B Coley, Cana L Brown, Michael H Shaw, Sayema Naaz, Ayush Dahal, Shahem Y Alqudah, Kevin N Nguyen, Anita D Nguyen, Sunita S Paudel, John E Shell, Suhas S Patil, Hong Dang, Wanda K O’Neal, Michael R Knowles, Dominika Houserova, Mark N Gillespie, Glen M Borchert

**Affiliations:** Department of Pharmacology, University of South Alabama, 5795 USA Drive North, Mobile, AL 36688, USA; Department of Pharmacology, University of South Alabama, 5795 USA Drive North, Mobile, AL 36688, USA; Department of Pharmacology, University of South Alabama, 5795 USA Drive North, Mobile, AL 36688, USA; Department of Pharmacology, University of South Alabama, 5795 USA Drive North, Mobile, AL 36688, USA; Department of Pharmacology, University of South Alabama, 5795 USA Drive North, Mobile, AL 36688, USA; Department of Pharmacology, University of South Alabama, 5795 USA Drive North, Mobile, AL 36688, USA; Department of Pharmacology, University of South Alabama, 5795 USA Drive North, Mobile, AL 36688, USA; Department of Pharmacology, University of South Alabama, 5795 USA Drive North, Mobile, AL 36688, USA; Department of Engineering, University of South Alabama, 150 Student Services Drive, Mobile, AL 36688, USA; Department of Biomedical Sciences, University of South Alabama, 5721 USA Drive North, Mobile, AL 36688, USA; Department of Biomedical Sciences, University of South Alabama, 5721 USA Drive North, Mobile, AL 36688, USA; Department of Biomedical Sciences, University of South Alabama, 5721 USA Drive North, Mobile, AL 36688, USA; Department of Pharmacology, University of South Alabama, 5795 USA Drive North, Mobile, AL 36688, USA; Department of Pharmacology, University of South Alabama, 5795 USA Drive North, Mobile, AL 36688, USA; Department of Pharmacology, University of South Alabama, 5795 USA Drive North, Mobile, AL 36688, USA; Marsico Lung Institute, University of North Carolina at Chapel Hill School of Medicine Cystic Fibrosis/Pulmonary Research & Treatment Center, 125 Mason Farm Road, Chapel Hill, NC 27599-7248, USA; Marsico Lung Institute, University of North Carolina at Chapel Hill School of Medicine Cystic Fibrosis/Pulmonary Research & Treatment Center, 125 Mason Farm Road, Chapel Hill, NC 27599-7248, USA; Marsico Lung Institute, University of North Carolina at Chapel Hill School of Medicine Cystic Fibrosis/Pulmonary Research & Treatment Center, 125 Mason Farm Road, Chapel Hill, NC 27599-7248, USA; Center for Cellular and Molecular Therapeutics at Children’s Hospital of Philadelphia, 3501 Civic Center Boulevard, Philadelphia, PA 19104, USA; Department of Pharmacology, University of South Alabama, 5795 USA Drive North, Mobile, AL 36688, USA; Department of Pharmacology, University of South Alabama, 5795 USA Drive North, Mobile, AL 36688, USA

## Abstract

Several studies have now described instances where G-rich sequences in promoters and enhancers regulate gene expression through forming G-quadruplex (G4) structures. Relatedly, our group recently identified 301 long genomic stretches significantly enriched for minimal G4 motifs (LG4s) in humans and found the majority of these overlap annotated enhancers, and furthermore, that the promoters regulated by these LG4 enhancers are similarly enriched with G4-capable sequences. While the generally accepted model for enhancer:promoter specificity maintains that interactions are dictated by enhancer- and promoter-bound transcriptional activator proteins, the current study tested an alternative hypothesis: that LG4 enhancers interact with cognate promoters via a direct G4:G4 DNA-based mechanism. This work establishes the nuclear proximity of LG4 enhancer:promoter pairs, biochemically demonstrates the ability of individual LG4 single-stranded DNAs (ssDNAs) to directly interact target promoter ssDNAs, and confirms that these interactions, as well as the ability of LG4 enhancers to activate target promoters in culture, are mediated by G4 DNA.

## Graphical Abstract

**Graphical Abstract:**
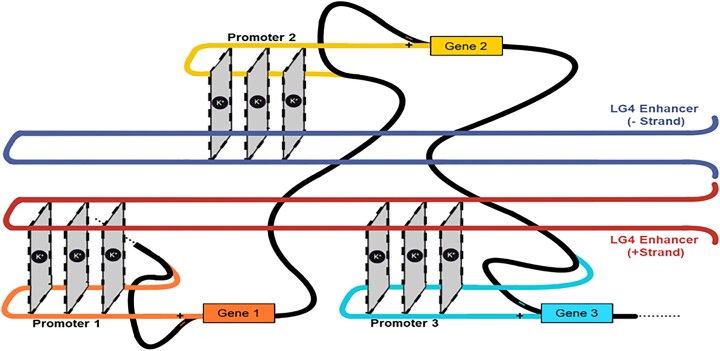

## Introduction

Enhancers are genomic sequences that function as *cis-*acting regulatory elements capable of increasing the transcriptional activity of a given gene(s) by recruiting transcription factors (TFs) to a target promoter (1). Enhancers are often located at a considerable distance from target promoters, and the broadly accepted model of enhancer activation is that an activator protein complex assembles on an enhancer before being transferred to the target promoter (2). First proposed in prokaryotes in 1986, the looping model of enhancer:promoter communication (3) (in which looping of the DNA separating an enhancer and its target promoter brings these regulatory elements into close proximity) is now almost universally accepted (1). Notably, the development of methods to interrogate the spatial organization of the genome via proximity ligation has helped firmly establish this mechanism as the predominant form of enhancer:promoter communication in vertebrates (4). As an example, a recent micro-C analysis confirmed that >65% of experimentally verified enhancer:promoter pairs associate through enhancer:promoter looping in human K562 lymphoblast cells (5). In addition, the ability of enhancers and promoters to contact one another is also partially controlled through organizing chromosomes into topologically associated domains (TADs) which often directly limits the range of enhancer influence (6–9). That said, enhancers frequently demonstrate selectivity for specific promoters within individual TADs with enhancers routinely crossing other intervening genes that are not activated by the enhancer during the ‘search’ for a target promoter (2,10,11). Several models have been proposed to explain promoter selectivity, but to date, the mechanisms responsible for the majority of specific enhancer:promoter interactions remain unresolved.

**Figure 1.**
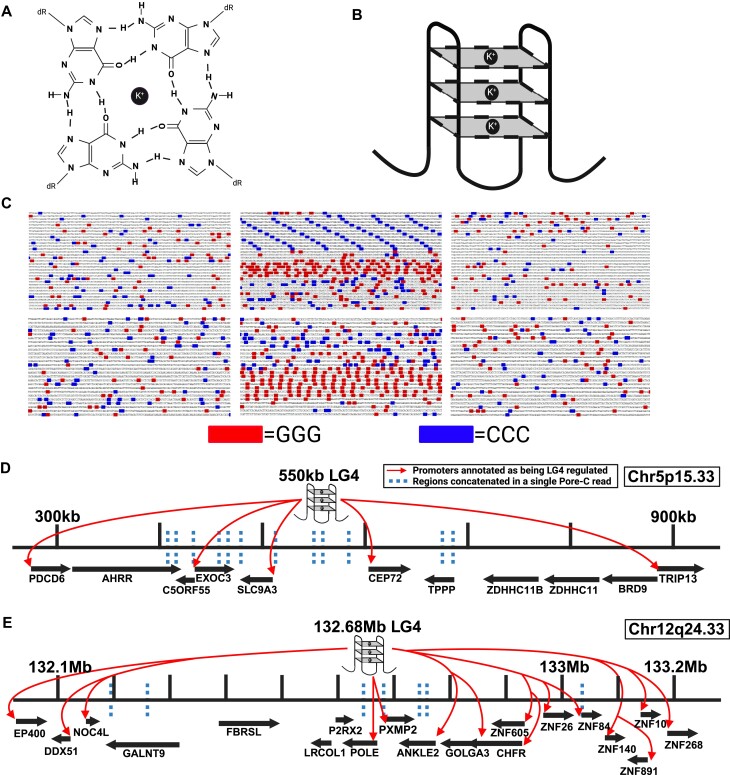
LG4 DNA. (**A**) Structure of an individual G-quartet wherein guanine nucleotides are held together by hydrogen bonds centered about a central potassium cation (K+). (**B**) Cartoon depiction of unimolecular antiparallel G4 DNA where each square corresponds to an individual G-quartet and the corners of each quartet correspond to a single guanine. (**C**) Two examples of LG4 loci in which ≥3 consecutive genomic Gs and ≥3 consecutive genomic Cs are highlighted. Top center, LG4 located at human Chr5:551935:556936:1; Top left and right, ∼5 kb sequences located 100 kb upstream and downstream of the central LG4 (Chr5:451935:456936:1 and Chr5:651936:656935:1, respectively). Bottom center, LG4 located at human Chr12:132686134:132690031:1. Bottom left and right, ∼5 kb sequences located 50 kb upstream and downstream of the central LG4 (Chr12:132636134:132640031:1 and Chr12:132736134:132740031:1, respectively). (**D**) Illustration of an insulated neighborhood within Chr5p15.33 centered on the LG4 located at Chr5:551935:556936:1 which is depicted above the assembly as a cartoon G4 structure labeled 550kb LG4. Protein coding genes and their orientation as annotated in Ensembl (25) are indicated by arrows located below the line segment representing the specified insulated neighborhood. Arrows originating at the LG4 and ending at various gene promoters indicate GeneHancer annotated promoter regulations for enhancers within the LG4 locus (GH05J000553, GH05J000555). Dashed lines represent regions of this insulated neighborhood which were concatenated to the LG4 within a single Pore-C read (Read SRR11589412.3086865.1). (**E**) Illustration of an insulated neighborhood within Chr12q24.33 which contains an LG4 located at Chr12:132686134:132690031:1. The LG4 is shown as a cartoon G4 structure labeled 132.68Mb LG4. Arrows originating at the LG4 and terminating at various promoters denote GeneHancer annotated promoter regulations (GH12J132686). The dashed lines denote regions of this insulated neighborhood which were joined to the LG4 within a single Pore-C read (Read SRR11589401.9930809.1). Genomic positions refer to GRCh38. Annotations of Pore-C reads (SRR11589412.3086865.1 and Read SRR11589401.9930809.1) are detailed in Supplementary Table S1.

Equally relevant to the work described herein, roles for guanine rich repetitive sequences in transcriptional regulation are now widely accepted (12–14). Non-coding guanine (G)-rich sequences are commonly found in gene promoters and enhancers, and several independent studies have described specific instances where G-rich repetitive sequences regulate gene expression via their capacity to form transient G-quadruplexes (G4s) under physiological conditions (15). G4s are non-B form DNA secondary structures that arise from Hoogsteen base pairing between G nucleotides stabilized by a central cation (16) (Figure 1A). While there is a large degree of heterogeneity among sequences capable of forming G4s, the minimally defined sequence thought to be required for G4 formation consists of four adjacent runs of G triplets separated by a spacer sequence of varying length (GGGnGGGnGGGnGGG) (16,17) (Figure 1B). Search algorithms modeled after variations of this minimal G4 motif routinely identify hundreds of thousands of putative G4 capable sequences in the human genome (18–20), although the true number of G4 forming loci, as well as the timing and cellular circumstances during which G4 structures actually form, have yet to be fully described.

Instead of focusing on shorter, more rigidly defined G4 motifs, our group recently searched the human genome for long genomic stretches significantly enriched for minimal G4 motifs (referred to as LG4s herein). Since G-rich immunoglobulin switch regions (21,22) are composed of a high percentage of (and routinely form) G4 structures and are characteristically associated with DNA breaks and altered gene expressions (21,22), we modeled our search parameters on the G-rich Sμ immunoglobulin switch region. Using this strategy, we previously identified 301 LG4 loci with a density of at least 80 GGG repeats/1000 bp and averaging 1843 bp in length (see examples in Figure 1C) (23). Further, in agreement with previous reports indicating that minimal G4s are highly enriched in promoters and enhancers (13,14), we found LG4s (in comparison to size matched control loci) significantly associated with transcriptional regulatory elements (24,25) and enriched in 26 different TF Chromatin Immunoprecipitation-sequencing (ChIP-seq) datasets (26,27), including CTCF, which is known to facilitate enhancer:promoter interactions and long-distance enhancer-dependent transcription (28). Furthermore, and of particular relevance to this study, we also found 217 of 301 LG4 sequences overlap a GeneHancer (29) annotated enhancer, and our initial analyses of the gene promoters regulated by these LG4 enhancers (as indicated by GeneHancer) found these promoters similarly, markedly enriched with G4-capable sequences (23).

Importantly, while the current generally accepted model for enhancer:promoter communication involves initial targeting of specific sequences in genomic enhancers and promoters by transcriptional activator proteins followed by enhancer:promoter engagement being facilitated by interactions between these proteins (1,11), our observations pointed to an alternative hypothesis; namely, that LG4 enhancers physically interact with their cognate promoters via a direct G4:G4 DNA based mechanism. Accordingly, in this work we employ a combination of informatic mining and locus-specific immunoprecipitation strategies to establish the spatial proximity of interacting loci within the nucleus then biochemically assess the ability of LG4 DNA sequences to directly interact with DNA sequences embedded in their cognate promoters.

## Materials and methods

### Detailing LG4 enhancer overlaps, promoter G4 capacity and LG4 loci gene fusions

LG4 and gene promoter locations were taken as occurring in Human Genome Release 77 (hg38) (25). Potential enhancer regulations were obtained from: (i) Ensembl Regulatory Build (24), (ii) a comprehensive Super-Enhancer database (SEdb 2.0) (30), (iii) UCSC genome browser Encode annotations (31), and (iv) the GeneHancer DB (29). Statistical significance of LG4 overlaps with annotated human enhancer elements compared to size and nucleotide composition matched control loci were determined using an unpaired one-tailed *t*-test. Significant enrichment of potential G4 contributing sequences (e.g. GGG) in LG4 neighborhood promoters (or randomly selected, size matched promoters not proximal to an LG4) was calculated using chi-square after individual motifs (e.g. GGG) were enumerated using an in house python script. Full FusionGDB gene fusion datasets (32) were downloaded and fusions between genes in LG4 neighborhoods (or randomly selected, size matched control locations) identified then significance calculated using an unpaired two-tailed *t*-test [as in (23)].

### Analysis of Pore-C datasets

First, all > 100 bp sequences perfectly mapping to multiple places in the human genome (hg38) (25) occurring within promoters were masked and excluded from consideration. Resulting masked promoter sequences were then aligned to individual reads obtained from publicly available Pore-C datasets housed in the NCBI SRA database (26) via BLAST (33). BLAST parameters were set at 100 max target sequences, word size 15, and an expected alignment threshold of e value = 1 × 10^−15^. Match/mismatch scoring parameters were 2,−3. Gap costs were existence: 5 and extension: 2 and alignments to low complexity regions were allowed. The 100 highest scoring Pore-C reads aligning to each promoter in individual datasets were identified then aligned to the human genome using the blastn tool in the Ensembl genome browser (25) with search parameters set at 500 max target sequences, word size 11, and an expected alignment threshold of e value = 1 × 10^−50^. Scoring parameters consisted of match/mismatch scores (1,−3), and gap penalties enforced as opening: 2, extension 2. Alignments to low complexity regions were allowed and query sequences were not filtered using RepeatMasker. In the event that a portion of a Pore-C read aligned to more than one place, the alignment with the lowest e-value was considered the bonafide alignment. Individual reads containing sections of both an interacting LG4 and promoter were identified then the origin of each fragment contained within a given read similarly defined by genomic alignment (Supplementary Tables S1 and S4).

### Enhancer quadruplex immunoprecipitation

PC3 cells were grown to 100% confluence in a T75 flask then crosslinked with glutaraldehyde (Fisher Scientific Hampton, NH cat no. O2957-1) diluted to 1% in phosphate-buffered saline (PBS) (Gibco Waltham, MA cat no. 10010049). Crosslinking reactions were incubated for 10 min at room temperature (RT) while slowly mixing on an orbital shaker. Crosslinking was then quenched by adding 1 ml of 10× glycine (Thermo Fisher Scientific Waltham, MA cat no. 043497–36) then incubating 5 min at RT followed by 10 min on ice. At this point, crosslinked cells were either permeabilized for downstream procedures or flash frozen and stored at −80°C for future use. To permeabilize cells, they were resuspended in a mix of 500 μl of 4°C permeabilization buffer [10 mM Tris-HCl (pH 8.0) (Fisher Scientific cat no. 77–86-1), 10 mM NaCl (Fisher Scientific cat no. S25541A), 0.2% IGEPAL CA-630 (Sigma-Aldrich St. Louis, MO cat no. I3021)] supplemented with 50 μl protease inhibitor cocktail III (Thermo Fisher Scientific cat no. J64283-LQ) and incubating on ice for 15 min. Cells were then pelleted by centrifugation at 500 × *g* for 10 min and washed with 200 μl of 1.5× DpnII reaction buffer (New England Biolabs Ipswich, MA cat no. R0543S), pelleted again and then resuspended in 300 μl of cold 1.5× DpnII reaction buffer (NEB cat no. R0543S). Chromatin were denatured by adding 33.5 μl of 1% sodium dodecyl sulfate (SDS) (Fisher Scientific cat no. BP166-100) then incubated for exactly 10 min at 65°C with gentle agitation then placed immediately on ice. SDS was quenched by adding 37.5 μl of 10% Triton X-100 (Sigma-Aldrich cat no. 93443) then incubating on ice for 10 min. Cells were pelleted briefly (pulsed at 500 × *g*) to remove supernatant, then digested with a final concentration of 1 U/μl of DpnII (NEB cat no. R0543S) in 450 μl of 1× digestion reaction buffer. Reactions were gently mixed by inversion and incubated at 37°C for 18 h while shaking at 250 rpm to prevent condensation. The restriction digest was then heat inactivated by incubating at 65°C for 20 min. Cells were then pelleted by centrifugation at 500 × *g* for 1 min to remove supernatant and then resuspended in a complete T4 ligation reaction mix consisting of 100 μl 10× T4 ligase buffer, 10 μl 10 mg/ml bovine serum albumin (BSA) (NEB cat no. B9000s), 840 μl DNA grade H_2_O (Invitrogen Waltham, MA cat no. AM9922) and 50 μl T4 DNA Ligase (NEB cat no.M0202L). Ligation reactions were cooled to 16°C and incubated for 6 h. Upon completion of the ligation reaction, cells were reverse crosslinked by treatment with 100 μl (20 mg/ml) Proteinase K (Thermo Fisher Scientific cat no. FEREo0491), 100 μl 10% SDS (Fisher Scientific cat no. BP166-100) and 500 μl 20% Tween-20 (Fisher Scientific cat no. BP337-500) in a total volume of 2000 μl with nuclease free water (Invitrogen cat no. AM9922) and incubated at 56°C for 18 h. After reverse crosslinking, samples were pelleted by centrifugation at 16 000 × *g* for 1 min and resuspended in complete lysis buffer consisting of 20 μl protease cocktail inhibitor III (Thermo Scientific cat no. J64283-LQ), 5 μl RNase A (Invitrogen cat no. 46–7604), 1000 μl lysis buffer [50 mM Tris-HCl (pH 7.0) (Fisher Scientific cat no. 77–86-1), 10 mM ethylenediaminetetraacetic acid (EDTA) (Fisher Scientific cat no. S311-500), 1% SDS (Fisher Scientific cat no. BP166-100)]. If no pellet is observed after centrifugation of reverse-crosslinking reaction, then proceed with hybridization using the reverse crosslinking reaction mix in place of lysis buffer; 1.7 ml hybridization buffer [750 mM NaCl (Fisher Scientific cat no. S25541A), 50 mM Tris-HCl (pH 7.0) (Fisher Scientific cat no. 77–86-1), 1 mM EDTA (Fisher Scientific cat no. S311-500), 1% SDS (Fisher Scientific cat no. BP166-100)] supplemented with 300 μl formamide (Fisher Scientific cat no. BP227-500) and 10 μl RNase A (Invitrogen cat no. 46–7604) was added for every 1 ml of cell lysate (or reverse crosslinking reaction). A total of 100 pM of Biotinylated probes (Integrated DNA Technologies Coralville, IA) antisense to the Chr5LG4 (primer probe table) or non-targeting control probes (or no probes for input DNA control) were added to the hybridization mix and incubated at 37°C for 4 h in an orbital shaker (250 rpm) following this step the sample designated for input DNA was isolated by phenol-chloroform isoamyl alcohol 25:24:1 (Invitrogen cat no. 15593–031) extraction. To perform streptavidin pulldown, 120 μl (4 mg/ml) streptavidin magnetic beads (NEB cat no. 50–812-660) were washed three times with 1 ml of lysis buffer then resuspended in 100 μl of complete lysis buffer and added to the hybridization mix and then incubated at 37°C for an additional 30 min with mixing. Tubes were placed on a magnetic separator until solution was clear. Supernatant was aspirated and streptavidin magnetic beads were washed once with 1 ml of pre-warmed wash buffer consisting of 2× Saline-Sodium Citrate (SSC) (Thermo Fisher Scientific cat no. AM9763), 0.5% SDS (Fisher Scientific cat no. BP166-100), 1 mM 4-(2-aminoethyl)benzenesulfonyl fluoride hydrochloride (Fisher Scientific cat no. AC328110500) supplemented with 20 μl protease cocktail inhibitor III (Thermo Scientific cat no. J64283-LQ). Tubes were placed back on magnet until solution became clear, the supernatant was discarded and the DNA was eluted from the magnetic beads by adding 150 μl of DNA grade H_2_O (Invitrogen cat no. AM9922) and incubating at 37°C for 30 min while shaking (250 rpm). To maximize yield the elution step can be repeated a second time. DNA was quantified by Qubit (Thermo Fisher scientific cat no. Q33238) and used as template DNA for polymerase chain reaction (PCR) to verify genomic interactions with the Chr5 LG4. If eluted DNA is insufficiently pure, a subsequent extraction by phenol-chloroform isoamyl alcohol 25:24:1 can be performed. PCRs were performed using primers specific to regions suspected to interact with the Chr5 LG4 or to control regions not predicted to interact with the Chr5 LG4. The primer sequences used in each reaction can be found in the primer probe table. PCRs were performed in 50 μl reactions with 1 μl (10 μM) forward primer, 1 μl (10 μM) reverse primer, 3 μl MgCl_2_ (25 mM), 2 μl DNTP (10 mM each), 5 μl 10× Taq buffer, 1.25 μl Taq polymerase (1 U/μl) (Fisher Scientific cat no. FEREP0404) and 61 pg of enhancer quadruplex immunoprecipitation (EQuIP) DNA or 10 ng input DNA filled to a 50 μl with DNA grade H_2_O (Invitrogen cat no. AM9922). The thermal cycling parameters for these reactions were an initial denaturation at 95°C for 1 min, followed by 32–37 cycles of 30 sec at 95°C, 30 sec at 57°C, 25 sec at 72°C, then a final extension step at 72°C for 1 min. PCR products were then run on a 1% agarose Tris Borate EDTA (TBE) gel for 50 min at 100 V and stained with EtBr (Fisher Scientific cat no. BP102-5).

### Electrophoretic mobility shift assays

All LG4 and promoter elements used in electrophoretic mobility shift assays (EMSAs) were cloned into TOPO TA pCR2.1 (Invitrogen cat no. 45–064-1) in both the sense and antisense orientations and verified by sequencing. Confirmed constructs were transformed into F’Iq Competent E. coli (NEB cat no. C2992H) and resulting bacterial colonies used to inoculate 50 ml of LB and grown at 37°C for 6 h at 250 rpm in an orbital shaker. Next, M13KO7 helper phage (NEB cat no. N0315S) was added to each culture (final concentration of 1 × 10^8^ pfu/ml) and incubated at 37°C for 1.5 h at 250 rpm after which kanamycin was added (final concentration 70 μg/ml) then grown overnight at 37°C at 250 rpm. Single-stranded DNA (ssDNA) was isolated the following day per M13KO7 helper phage standard manufacturer protocol (NEB cat no. N0315S).

ssDNA constructs generated by M13KO7 helper phage were run on a 1% agarose 1× TBE gel at 100 V for 45 min then size selected ssDNA purified by gel extraction using Wizard SV Gel and PCR Clean-Up System (Promega, Madison, WI, cat no. PR-A9281) and quantified via Nanodrop 6000 (Thermo-Scientific). A total of 20 μl (15 ng/μl) of each ssDNA promoter were boiled either separately or together with 10 μl (10 ng/μl) of LG4 ssDNA (for samples containing only LG4, 20 μl of LG4 ssDNA was used) at 98°C for 10 min then held at 80°C for 10 min during which pre-heated KCl solution (to fold G4) [final (250 mM)] or an equal volume of pre-heated ultrapure H_2_O (unfolded controls) was added to the indicated samples and slow cooled to 45°C over 1 h then held at 16°C. Resulting ssDNAs folded in either KCl or water were next ran on a 1.5% agarose 1× Tris-glycine (Bio-Rad Hercules, CA, cat no. 1610734) gel at 75 V for 8 h at 4°C after which gels were stained for 24 h with SYBR Gold (Invitrogen cat. S11494) diluted 1:10000 in 1× Tris-glycine (Bio-Rad cat no. 1610734) then imaged on a UV Transilluminator FBTIV-88 (Fisher Scientific). Specified gels were stained at 4°C for 2 h with N-methyl mesoporphyrin IX (Fisher Scientific, cat no. 50–385-23) diluted in 1× Tris-glycine to achieve a final concentration of 2 μM followed by an additional 6 h stain at 4°C in a 1× Tris-glycine solution supplemented with N-methyl mesoporphyrin IX (50 μM final concentration). Following this the same gel was stained overnight with SYBR Gold diluted 1:10000 in 1× Tris-glycine as previously described.

### Plasmid construction

*Topo TA pCR2.1 plasmids*. The EXOC3 promoter, GOLGA3 promoter, EP400 Promoter, HIF1A promoter, SV40 control enhancer, Chr5LG4 and Chr12 LG4 were each PCR amplified using 10 ng of WI2-3695P19 fosmid DNA, WI2-3322N15 fosmid DNA, WI2-997I12 fosmid DNA, pGL4.20-HIF1A prom plasmid DNA (used for HIF1A promoter and SV40 control enhancer), WI2-1251C21 fosmid DNA and WI2-3035P11 fosmid DNA as templates, respectively, whereas the ZNF84 and CEP72 promoters were PCR amplified using 10 ng of PC3 DNA as template. Deletion constructs were amplified from plasmids containing full length promoters. Fosmids were obtained from BACPAC genomics resource center (BACPAC Genomics, Inc. Redmond, WA) and isolated by HighPrep Plasmid DNA Kit (Magbio Genomics Inc. Gaithersburg, MD cat no. 501656596) whereas the pGL4.20-HIF1A prom plasmid was a gift from Alex Minella (Addgene plasmid # 40173; http://n2t.net/addgene:40173;RRID:Addgene_40173) and isolated by Zyppy Plasmid Miniprep Kit (Zymo Research Irvine, CA cat no. D4020). All PCRs were performed using LongAmp Taq DNA polymerase (NEB, cat no. 50994936) in a 25 μl reaction volume according to manufacturer’s protocol. Resulting amplicons were purified by gel extraction using Wizard SV Gel and PCR Clean-Up System (Promega cat no. PR-A9281) then cloned into TOPO TA pcR2.1 (Invitrogen cat no. 45–064-1). All primers utilized to generate each construct are listed in Supplementary Table S6. Of note, nested PCRs were ultimately necessary to clone the GOLGA3 promoter and chr12 LG4. For constructs amplified by nested PCR, the primers listed in Supplementary Table S6 are numbered to indicate reaction order. All resulting clones were verified by sequencing (Eurofins USA Lancaster, PA).

*pGL4-based reporter plasmids*. The EXOC3 promoter reporter was constructed by PCR amplification of the EXOC3 promoter sequence using the previously constructed Topo-EXOC3 plasmid as DNA template and TOPO TA pcR2.1 specific primers tagged with NheI and BglII restriction sites on the forward and reverse primers, respectively. The resulting amplicon was gel purified with Wizard SV Gel and PCR Clean-Up System (Promega cat no. PR-A9281), digested with NheI (Thermo Scientific cat no. FD0974) and BglII (Thermo Scientific cat no. FD0083) restriction enzymes, followed by gel purification with Wizard SV Gel and PCR Clean-Up System (Promega cat no. PR-A9281) and subsequent T4 ligation (NEB cat no. M0202L) to the pGL4.10 backbone. The HIF1A promoter reporter was generated by restriction digest of pGL4.20 with BgII (Thermo Scientific cat no. FD0074) and HindIII (Thermo Scientific cat no. FD0504) restriction enzymes, followed by gel purification with Wizard SV Gel and PCR Clean-Up System (Promega cat no. PR-A9281) and subsequent T4 ligation (NEB cat no. M0202L) to the pGL4.10 backbone.

### Luciferase assays

A total of 5000 A549 lung cancer cells were seeded into a 96-well plate with an opaque bottom. Cells were allowed to adhere to the plate by incubating at 37°C for 2 h. Cells were then transfected with 50 ng of luciferase construct along with 50 ng of enhancer containing plasmid using Lipofectamine 3000 transfection reagent (Invitrogen cat no. L3000001) according to manufacturer’s protocol. Transfected cells were then incubated at 37°C for 24 h before being lysed in plate with an equal volume of One-Glo Luciferase reagent (Promega cat no. E6110). Reactions were incubated at RT for 3 min followed by measurement of luminescence using an LMAX II 384 microplate reader (Molecular Devices model no. 38100–33). LG4/SV40 ratios in each promoter cohort were obtained by dividing each relative light unit (RLU) value obtained from cotransfection of + or − orientation Chr5 LG4 by the average RLU of + or − orientation SV40 for that run. Luciferase assays were performed on three separate occasions to confirm reproducibility using Graphpad Prism software with an unpaired two-tailed *t*-test with Welsh’s correction. For select luciferase assays, the G4-binding agent, TMPyP4 (Millipore Sigma, cat. no 613560–25MG) [100 nM] was added to the cell culture media at the time of transfection and incubated with the cells for the entirety of the assay.

### Dot blotting

Dot blots were performed using Biodyne B nylon membranes (Thermo Scientific, cat no. 77016). Membranes were soaked in 1× Tris Buffered Saline (TBS) for 15 min followed by dotting of 20 μl (2.5 ng/μl) of ssDNA samples onto the membrane using the Bio-Dot Apparatus (Bio-Rad, cat no. 1706545). ssDNA was then fixed to the membrane by crosslinking twice for 5 min at 120 000 μJ/cm^2^ using an Ultraviolet Multilinker (Ultra Lum Inc., Cat no. UVC515). Membranes were washed by gently rocking in 10 ml of a wash buffer consisting of 1× TBS supplemented with 0.1% Tween-20 for 5 min at RT. The membranes were then incubated at RT for 1 h 10 min in a blocking buffer consisting of 1× TBS supplemented with 5% nonfat dry milk (BioKEMIX, cat no. M0841) and 1% BSA (Promega, cat no. W384A). The membranes were then incubated for 1 h at RT with the BG4 antibody (produced in-house as described below) (diluted 1:1000 in blocking buffer). Following this, the membranes were washed three times (once for 15 min and then twice for 5 min) with 10 ml of washing buffer. Membranes were then incubated for 1 h at RT with Rabbit anti-FLAG antibody (Proteintech, cat no. 20543–1-AP) diluted 1:800 in blocking buffer and then subjected to another 3× wash cycle as described previously. Then the membranes were incubated for 1 h at RT with Goat-anti Rabbit antibody (Proteintech, cat no. SA00001-2) diluted 1:2000 in blocking buffer. The membranes were subsequently washed four times (once for 15 min followed by three washes for 5 min). Chemiluminescent signal was determined using the SuperSignal West Pico PLUS Chemiluminescent Substrate (Thermo Scientific, cat no. 34579) according to the manufacturer’s recommendations and then imaged on a Bio-Rad ChemiDoc imaging system (Bio-Rad, cat no. 12003153).

BG4 antibody Production. The BG4 antibody used throughout this study is derived from the plasmid pSANG10-3F-BG4, which was given to our group as a gift from Shankar Balasubramanian (Addgene plasmid # 55756; http://n2t.net/addgene:55756;RRID:Addgene_55756). BL21 (DE3) Competent E.coli (NEB cat no. C2527H) were transformed with pSANG10-3F-BG4 and plated onto LB agar plates with Kanamycin (50 μg/ml) and grown at 37°C overnight. A colony was picked and grown in 2× YT medium supplemented with 50 μg/ml of Kanamycin at 37°C overnight. This overnight culture was then diluted (1:100) in fresh 2× YT media (50 μg/ml of Kanamycin) and grown for 3 h at 37°C while shaking at 250 rpm. Antibody expression was induced by supplementing the bacterial culture with Isopropyl β- d-1-thiogalactopyranoside (IPTG), in which a final concentration of 0.5 mM IPTG was achieved. The bacteria were then grown for an additional 4 h at 37°C while shaking at 250 rpm before being pelleted at 4000 × *g* for 30 min at 4°C. The cell pellet was then resuspended in 3 ml of Tris EDTA Sucrose (TES) Buffer (50 mM Tris-HCl pH 8.0, 1 mM EDTA, 20% (*w/v*) sucrose and 1× protease inhibitor (cat no. 50-488-957) and was incubated on ice for 10 min. The bacterial slurry was then diluted 2-fold in water and incubated on ice for an additional 10 min followed by centrifugation at 16000 × *g* for 30 min. The resulting supernatants were loaded onto HisPur™ Cobalt Spin Columns (Thermo Scientific, cat no. 89969), and gently rocked at RT for 30 min. The bottom plug was then removed from the filter and centrifuged at 700 × *g* for 2 min. The flow through was collected and the filter was washed twice with PBS supplemented with 10 mM Imidazole (Thermo Scientific, cat no. 288–32-4), collecting the flow through each time. Finally, the antibody was eluted three times with PBS supplemented with 250 mM Imidazole. The flow through from each wash step and elution step were concentrated using Amicon Ultra-15 Centrifugal Filters (MilliporeSigma, cat no. UFC901008).

## Results

### Chr 5 and Chr 12 LG4s are enhancers annotated as regulating G4-enriched promoters

After finding that 217 of the 301 LG4 loci identified in our initial study either fully or partially overlap with an annotated human enhancer compared to only 84 average overlaps (*n* = 5) (*P* < 0.00001) between size and nucleotide composition matched control loci and enhancers (23), we decided to select two LG4 loci (independently annotated as high confidence enhancers by multiple groups) for experimental validation. Notably, the first of these LG4s located at human chromosome 5p15.33 (Chr5:551935:556936) (Figure 1C and D) directly overlaps 13 reported enhancers independently annotated in the Ensembl regulatory build (24), the Encyclopedia of DNA Elements (ENCODE) (31), GeneHancer (29) and SuperEnhancer (34) datasets. Similarly, we also selected a second LG4 located at human chromosome 12q24.33 (Chr12:132686134:132690031) (Figure 1C and E) which directly overlaps nine reported enhancers independently annotated in Encode, GeneHancer and SuperEnhancer datasets (Table 1).

**Table 1.** LG4 enhancer annotations

Chromosome 5 LG4 enhancer annotations				
Enhancer ID	Source	Start position (Chr5)	Stop position (Chr5)	Noted gene association/regulations
GH05J000553	GeneHancer	553 660	554 453	EXOC3, CEP72, BRD9, SLC9A3
GH05J000555	GeneHancer	555 169	557 644	PDCD6, CEP72, BRD9, SLC9A3
SE_02_008600693	SEdb2.0	505 305	558 774	SLC9A3, LDC25845
SE_02_024100501	SEdb2.0	543 481	558 675	SLC9A3
SE_02_020700020	SEdb2.0	532 856	558 643	SLC9A3, CEP72
SE_00_002000529	SEdb2.0	498 817	557 908	SLC9A3, LOC25845
SE_00_003600317	SEdb2.0	498 742	557 880	SLC9A3, LOC25845
SE_02_035400733	SEdb2.0	473 202	554 505	SLC9A3, LOC25845, EXOC3, C5ORF55
ENSR00001256048	Ensembl	555 201	555 400	Not indicated
ENSR00001256049	Ensembl	556 401	556 600	Not indicated
EH38E2352151	ENCODE	553 896	554 206	SLC9A3, CEP72, EXOC3
EH38E2352153	ENCODE	555 051	555 387	SLC9A3, CEP72, EXOC3
EH38E2352157	ENCODE	556 903	557 118	SLC9A3, CEP72, EXOC3
	Chr5 LG4	551 935	556 936	
**Chromosome 12 LG4 enhancer annotations**				
**Enhancer ID**	**Source**	**Start position (Chr12)**	**Stop position (Chr12)**	**Noted gene association/regulations**
GH12Jl32686	GeneHancer	132 685 200	132 690 084	POLE, PXMP2, ANKLE2, NOC4L, EP400, ZNF891, ZNF84, ZNF140, GOLGA3, CHFR, ZNF605, DDX51, PGAM5, ZNF26, ZNF10
SE_02_116400065	SEdb2.0	132 684 308	132 695 135	PXMP2, POLE, PGAM5
SE_02_055500763	SEdb2.0	132 685 389	132 689 520	PXMP2, POLE, PGAM5
SE_02_039600318	SEdb2.0	132 688 822	132 789 737	ANKLE2, PXMP2, PGAM5, GOLGA3, POLE
SE_02_104301098	SEdb2.0	132 639 038	132 685 737	POLE, PXMP2, PGAM5, P2RX2, LRCOL1
EH38El65TT81	ENCODE	132 686 748	132 686 912	POLE, PXMP2, ANKLE2
EH38El657782	ENCODE	132 686 991	132 687 193	POLE, PXMP2, ANKLE2
EH38El657785	ENCODE	132 689 268	132 689 614	POLE, PXMP2, ANKLE2
EH38El65TT86	ENCODE	132 689 796	132 690 144	POLE, PXMP2, ANKLE2
	Chr12 LG4	132 685 134	132 690 031	

Enhancer positions overlapping Chr5 and Chr12 LG4s and their putative gene regulations were curated from GeneHancer, SEdb 2.0, Ensembl and ENCODE databases. LG4 positions are listed in red at the bottom of section.

In light of a model of G4-based enhancer:promoter interaction proposed in 2015 based on the identification of a propensity for single enhancer:promoter pairs to contain putatively interacting minimal G4 motif components (15), we next examined the potential of the promoters plausibly regulated by these LG4 enhancers to contribute to G4 formation. Analyses of the gene promoters likely regulated by LG4 enhancers find them significantly enriched with G4-capable sequences (23) suggesting that LG4 sequences could potentially form composite G4s with distal promoters in which a LG4 and interacting promoter each contribute a portion of the sequence necessary to form a composite G4. As an example, we find the average number of G triplets occurring on either strand within 5 kb upstream of the average human protein coding gene transcription start site (TSS) to be ∼142. In contrast, the average number of G triplets occurring within 5 kb upstream of the four genes (EXOC3, CEP72, BRD9, SLC9A3) annotated by GeneHancer (29) as being regulated by an enhancer wholly embedded within the Chr5 LG4 (GH05J000553) is notably higher at 237 (range 184–291) (Supplementary Table S2). Furthermore, we find the average number of G triplets available for contributing to composite G4 formation within a single LG4 to be 74-fold greater than the available G triplets in the average target-gene promoter (23) leading us to speculate that LG4 enhancers may act as long ‘Velcro-like’ regions simultaneously interacting with multiple gene promoters to coordinate their expressions (see ‘Graphical Hypothesis’).

### LG4s and putative target promoters are proximally located in the nucleus

**Figure 2.**
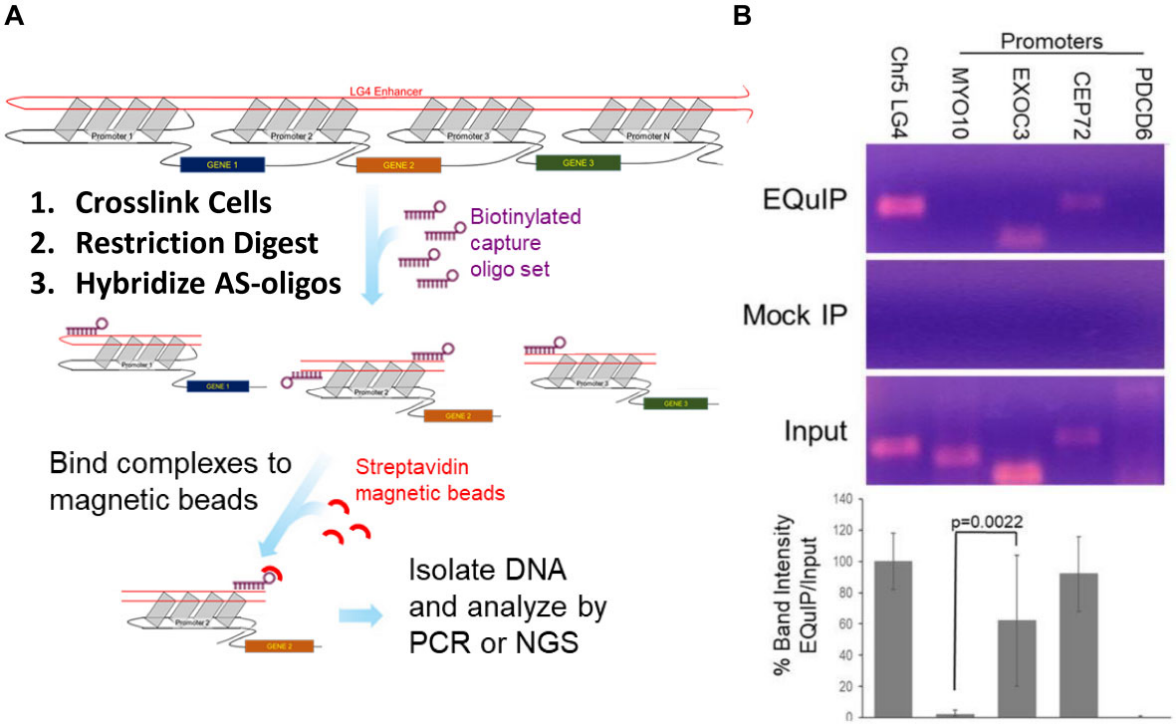
EQuIP (Enhancer Quadruplex ImmunoPrecipitation). (**A**) Cartoon of stepwise EQuIP protocol. In brief, EQuIP employs a probe set consisting of distinct biotinylated oligo probes complementary to different regions of the LG4. Probes are designed to target regions flanking sequences meeting the minimal criteria for G4 formation as flanking sequences are presumably held single stranded by their neighboring G4s and therefore free to basepair with complementary probes. These probe sets are combined with crosslinked, digested chromatin and allowed to hybridize to LG4 DNA. Complexes containing biotinylated-probes bound to LG4 DNA are isolated using streptavidin magnetic beads then DNA recovered and analyzed by PCR. (**B**) Representative PCRs employing DNA template isolated from PC3 cell EQuIP pulldowns, pulldowns using non-specific probes (Mock IP), or total input DNA collected prior to IP. PCR amplicons (∼300 bp) located: (Chr5 LG4) within the Chr5 LG4 or within the annotated promoters upstream of the primary TSS of the MYO10, EXOC3, CEP72 and PDCD6 protein coding genes. PCR amplicons were verified by sequencing. % band intensity (EQuIP IP/Input) is presented as a bar graph below corresponding amplicons and depicts average values and standard deviations corresponding to multiple independent EQuIP assay replicates (*n* ≥ 3). Significance determined by unpaired two-tailed *t*-test. Full gel images are shown in Supplementary Figure S1.

We find several lines of evidence supporting the proposed colocalization of LG4s and their putative target promoters: (i) Chromatin conformation capture is a method used to investigate interactions between genomic loci that are not adjacent in the primary sequence. In this method, genomic DNA is first cross-linked (to preserve the spatial proximity of interacting loci) then restriction digested before being enzymatically ligated to concatenate sequences that are proximally located in the nucleus despite occurring at distinct, non-contiguous genomic positions (4). One of these methods, Oxford Nanopore Pore-C, routinely generates concatenated reads averaging > 10 000 nt in length and as such is particularly well-suited for revealing enhancer:promoter interactions (37). In light of this, we elected to mine several Pore-C datasets [publicly available via the NCBI SRA repository (26)] and readily identified numerous examples of individual Pore-C reads containing segments of both an LG4 enhancer and its annotated target promoters strongly supporting their spatial proximity in the nucleus (Supplementary Tables S1 and S4, and Figure 1D and E). (ii) The correlation between spatial proximity and frequency of chromosomal translocations at the scale of individual genes is well documented (38). That said, we find genes potentially regulated by Chr5 and Chr12 LG4s frequently engage in local gene fusions [annotated in FusionGDB (32)] further supporting their proximity in nuclear chromatin (Supplementary Table S5). And (iii) a technique recently developed by our lab, called EQuIP (Enhancer Quadruplex Immunoprecipitation), directly confirms enrichments for individual gene promoters in LG4-specific immunoprecipitations (IPs) (Figure 2). Notably, we find EQuIP pulldowns of the Chr5 LG4 enriched for DNA from both the EXOC3 and CEP72 promoters [both of which are annotated as being regulated by an enhancer embedded in this LG4 (GH05J000553) (29)]. Importantly, Chr5 LG4 EQuIP analyses demonstrate clear enrichments of EXOC3 and CEP72 promoter sequences in pulldowns using probes targeting the Chr5 LG4 enhancer whereas neither sequences (39) (i) corresponding to nearby promoters not annotated as being regulated by this enhancer (the MYO10 promoter located ∼15 Mbp downstream and PDCD6 promoter located ∼300 kb upstream) (Figure 2B) nor (ii) located within the local Chr5 LG4 neighborhood but not occurring within an annotated promoter (AHRR 3′ intron) (Supplementary Figure S1) were appreciably enriched.

### DNA sequences within specific promoters and the Chr5 LG4 can physically interact

**Figure 3.**
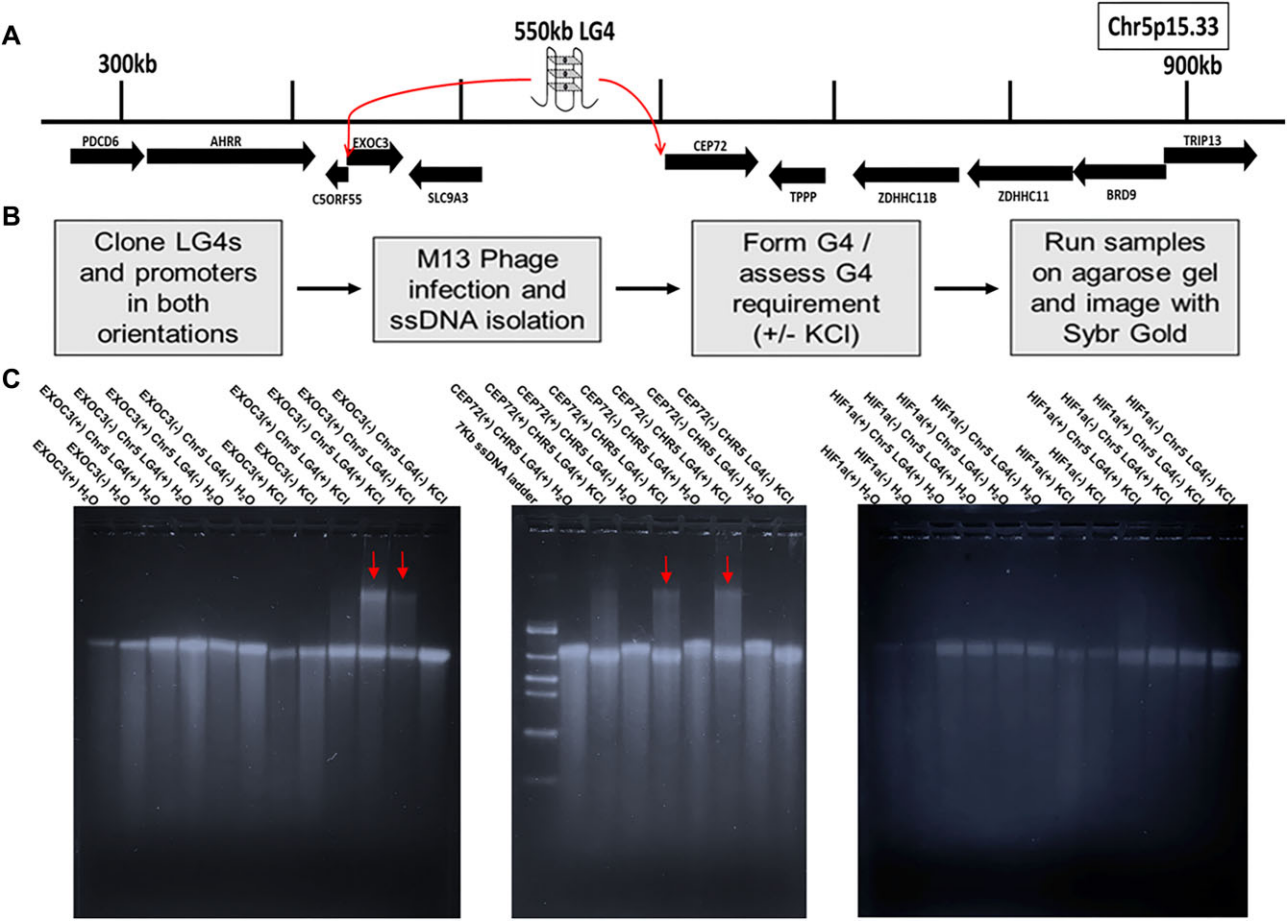
Direct interaction between EXOC3 and CEP72 promoters and the Chr5 LG4 enhancer. (**A**) Illustration of an insulated neighborhood within Chr5p15.33 centered on the LG4 located at Chr5:551935:556936 which is depicted above the assembly as a cartoon G4 structure labeled 550kb LG4. Protein coding genes and their orientation as annotated in Ensembl (25) are indicated as arrows below the line segment representing the genomic region. Arrows originating at the LG4 and ending at various gene promoters indicate GeneHancer annotated promoter regulations for enhancers within the LG4 locus (GH05J000553, GH05J000555). (**B**) Flow chart of EMSA protocol. (**C**) Images of 1.5% agarose Tris-glycine gels ran at 4°C for 8 h at 75 V and then stained for 24 h with SYBR Gold. Each construct was run on a gel in either the unfolded (H_2_O) or folded (KCl)[250 mM] state as indicated above the image. Samples including more than one construct were folded together. Red arrows denote gel shifts observed when a promoter and the Chr5 LG4 are folded together. See Supplementary Figure S2 for a side by side comparison of HIF1 and EXOC3, and Supplementary Figure S3 for detailed EMSA quantifications.

EMSAs were used to directly assess whether specific promoters can physically, independently interact with the Chr5 LG4 (Figure 3). Phagemids containing the EXOC3, CEP72 and HIF1A (a similarly sized, G4-capable promoter located on human Chr14) (40) promoters and the Chr5 LG4 were constructed and M13KO7 helper phage used to generate ssDNAs corresponding to each. Importantly, while no interactions between the G4-capable HIF1A promoter nor EXOC3 or CEP72 promoters and Chr5 LG4 ssDNAs were observed following incubation in non-G4-permissive conditions (H_2_O lacking KCl), a substantial, additive gel shift was clearly detected between ssDNAs corresponding to the + strand of the EXOC3 promoter and the − strand of the Chr5 LG4 when folded together under G4 permissive conditions (Figure 3C, left). Interestingly, a similar gel shift was also observed between ssDNAs corresponding to the − strand of the EXOC3 promoter and the + strand of the Chr5 LG4 whereas no interactions were observed between the − strand of the EXOC3 promoter and the − strand of the Chr5 LG4 nor the + strand of the EXOC3 promoter and the + strand of the Chr5 LG4 when incubated under G4 permissive conditions (Figure 3C, left). Of note, each phagemid generated ssDNA migrated faster after being incubated in G4 permissive (+KCl) versus non-permissive (H_2_O) conditions, regardless of their ability to independently form G4 DNA (Supplementary Figure S2), indicating KCl concentration alone can alter gel mobility [consistent with previous reports (41)]. That said, as the size of the observed gel shift closely approximates the predicted size of an intermolecular interaction between EXOC3 promoter and Chr5 LG4 ssDNAs (Supplementary Figure S2), these results strongly suggest that DNA sequences within the EXOC3 promoter and Chr5 LG4 directly interact with one another when coincubated in G4-permissive conditions.

Closely resembling shifts involving the EXOC3 promoter, a substantial, additive gel shift was clearly detected between ssDNAs corresponding to the + strand of the CEP72 promoter and the − strand of the Chr5 LG4 when folded together under G4 permissive conditions (Figure 3C, middle). Further resembling EXOC3 shifts, a similar gel shift was also observed between ssDNAs corresponding to the − strand of the CEP72 promoter and the + strand of the Chr5 LG4 whereas no interactions were observed between the − strand of the CEP72 promoter and the − strand of the Chr5 LG4 nor the + strand of the CEP72 promoter and the + strand of the Chr5 LG4 when incubated under G4 permissive conditions (Figure 3C, middle). Of note, in several instances smearing potentially indicating the formation of an array of less specific, higher molecular interactions was observed (e.g. lane containing the + strand of the CEP72 promoter and the + strand of the Chr5 LG4). As such, legitimate gel shifts were distinguished from non-specific interactions via generating detailed quantifications of individual lane band intensities by ImageJ analysis (Supplementary Figure S3).

Importantly, whereas HIF1A promoter DNA has previously been shown to independently form G4 structures (40), in contrast to EXOC3 and CEP72 promoter ssDNAs, we find no evidence of an interaction between HIF1A promoter and Chr5 LG4 ssDNAs folded together under G4 permissive conditions (Figure 3C, right). We suggest this likely supports that (i) G4 formation is necessary but not sufficient for G4-mediated enhancer:promoter interactions, and (ii) additional sequence complementarities (potentially involved with mediating enhancer:promoter interaction specificity) are likely required.

### Specific sequences within the EXOC3 promoter facilitate interaction with the Chr5 LG4

**Figure 4.**
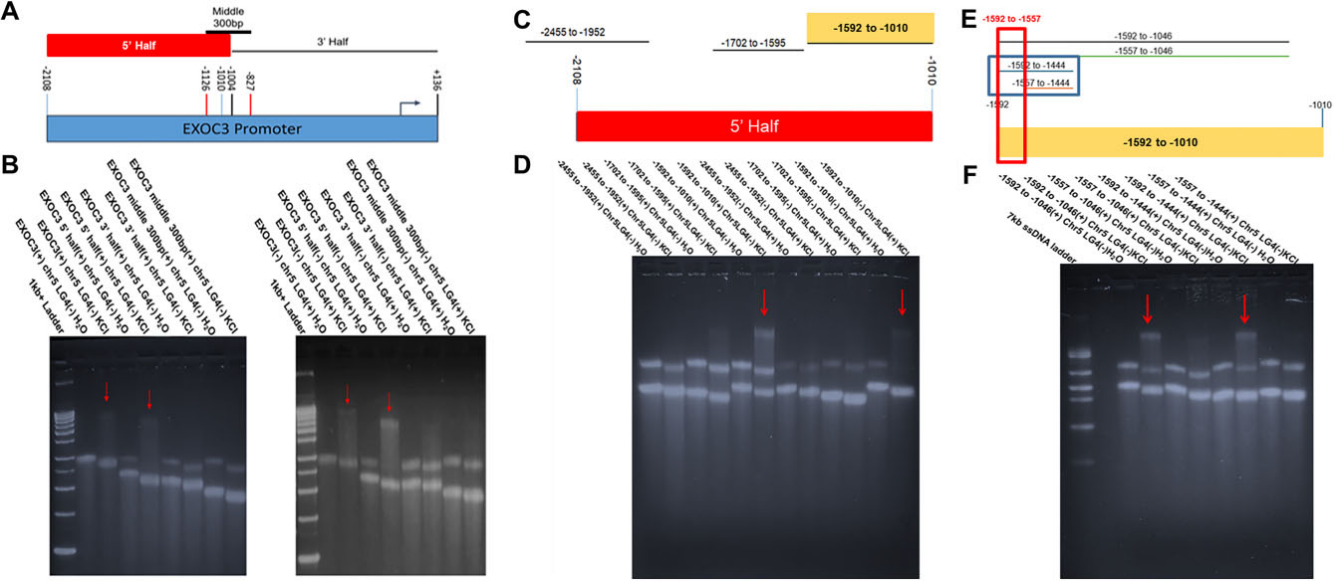
EXOC3 promoter deletion analysis. (**A**) Cartoon depiction of positions of deletion constructs within the full length EXOC3 promoter sequence. Nucleotide positions are given with respect to the TSS indicated by an arrow. (**B**) Images of 1.5% agarose Tris-glycine gels ran at 4°C for 8 h at 75 V then stained with SYBR Gold for 24 h. Sample content within each well is indicated above the gel image. The strand of each construct used in this EMSA is denoted as either (+) for the sense strand or (−) for the antisense strand. Each construct was run on a gel in either the unfolded (H_2_O) or folded (KCl)[250 mM] state as indicated above the image. (**C**) Cartoon depiction of positions of deletion constructs within the EXOC3 5′ Half promoter sequence identified in **B**. (**D**) Gel images generated identically to those in panel (B). (**E**) Cartoon depiction of positions of deletion constructs within the EXOC3 −1592 to −1010 promoter sequence identified in panel (D). (**F**) Gel images generated identically to those in panel (B). Arrows denote gel shifts observed when a promoter sequence and the Chr5 LG4 are folded together. See Supplementary Figure S3 for detailed EMSA quantifications.

In an attempt to better define the sequences involved with mediating the observed EXOC3:Chr5 LG4 interactions, we generated a series of truncated EXOC3 promoter constructs (Figure 4A) and found that a 982 bp portion of the EXOC3 promoter located between 2108 and 1126 bp upstream of the primary EXOC3 TSS is independently capable of interacting with the Chr5 LG4 (Figure 4B). Interestingly, although several minimal G4-capable sequences are located between the TSS and this region (TSS to 1126 upstream), the 982 bp mediating the interaction with the Chr5 LG4 is entirely devoid of any traditional G4-capable motifs (Supplementary Figure S4). As this clearly argues against interactions between G4s independently formed in both of the contributing sequences, we suggest these results instead agree with the general model proposed in our ’Graphical Hypothesis’ in which LG4 enhancers physically interact with gene promoters by forming composite G4 structures where both the LG4 and cognate promoter contribute a portion of the necessary sequence for G4 formation. Next, to further define the sequences involved with mediating the observed EXOC3:Chr5 LG4 interactions, we generated a series of truncated EXOC3 promoter constructs consisting of distinct sections of the 982 bp interacting portion of the EXOC3 promoter located between 2108 and 1126 bp upstream of the TSS (Figure 4C) and successfully further refined the minimally interacting sequence to a region located between −1592 to −1010 upstream of the EXOC3 TSS (Figure 4D). Next, successive deletion of the −1592 to −1010 interacting sequence (Figure 4E) identified a 148 bp portion of the EXOC3 promoter located between −1592 and −1444 bp upstream of the EXOC3 TSS independently capable of interacting with the Chr5 LG4 (Figure 4F). Interestingly, the ability of this 148 bp sequence to interact with the Chr5 LG4 was only lost upon specific mutation of GGG/CCC motifs located in a 35 bp region between −1592 and −1557 bp upstream of the EXOC3 TSS indicating their requirement for and supporting their direct participation in facilitating the observed EXOC3:Chr5 LG4 interaction (Figure 4E and F, and Supplementary Figure S4).

### DNA sequences within specific promoters and the Chr12 LG4 can physically interact

**Figure 5.**
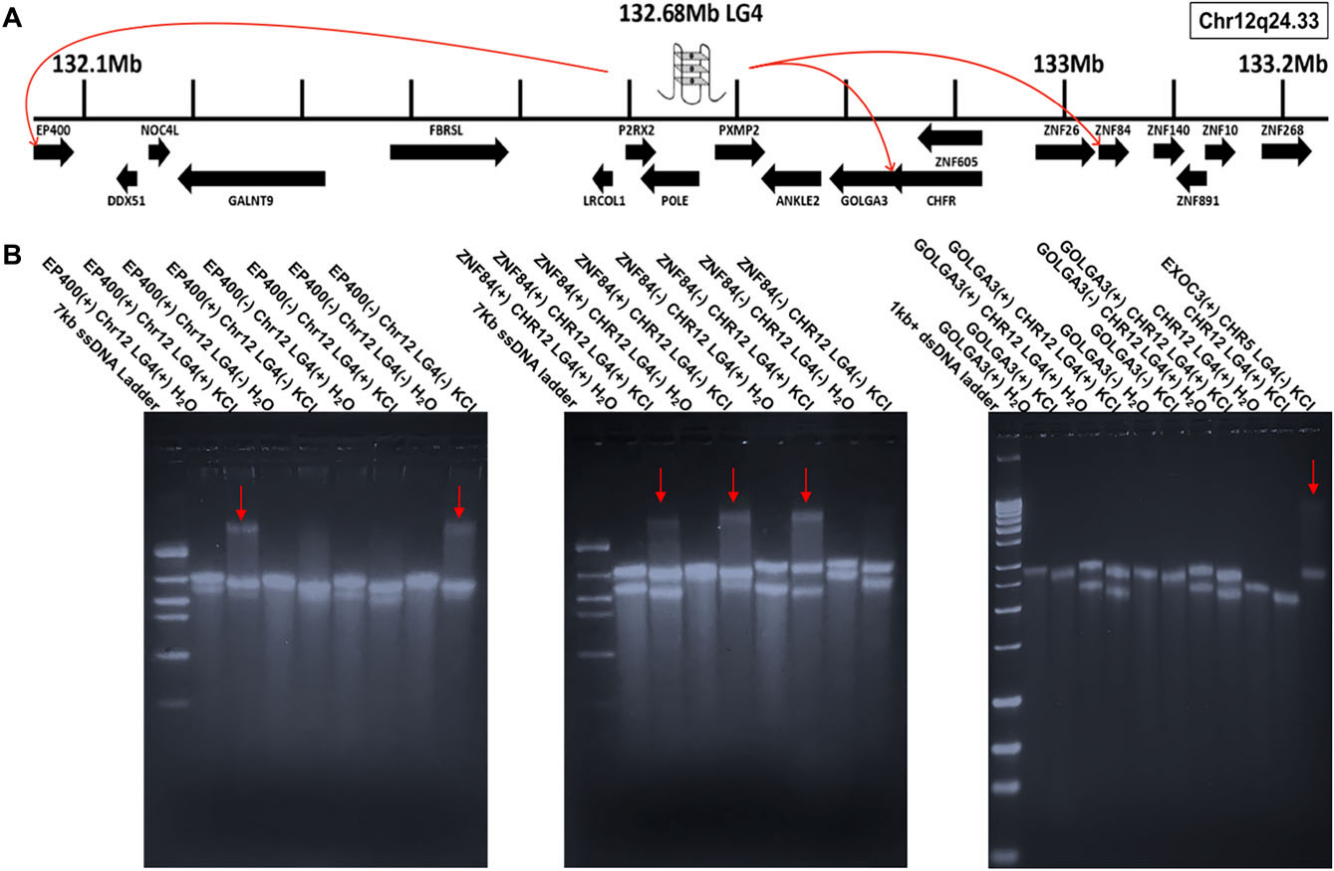
Direct interactions between EP400 and ZNF84 promoters and the Chr12 LG4 enhancer. (**A**) Illustration of an insulated neighborhood within Chr12q24.33 which contains an LG4 located at Chr12:132686134:132690031. The LG4 is shown as a cartoon G4 structure labeled 132.68Mb LG4. Arrows originating at the LG4 and terminating at various promoters denote GeneHancer annotated promoter regulations (GH12J132686). Protein coding genes and their orientation as annotated in Ensembl (25) are indicated as arrows below the line segment representing the genomic region. (**B**) Images of 1.5% agarose Tris-glycine gels ran at 4°C for 8 h at 75 V and then stained for 24 h with SYBR Gold examining EP400 (left), ZNF84 (middle) and GOLGA3 (right) promoters are shown. Each construct was run on a gel in either the unfolded (H_2_O) or folded (KCl)[250 mM] state as indicated above the image. Samples including more than one construct were folded together. Arrows denote gel shifts observed when a promoter and a LG4 are folded together. See Supplementary Figure S3 for detailed EMSA quantifications.

To determine if the ability of Chr5 LG4 ssDNAs to directly interact with specific promoters was unique to that locus, or instead, if other LG4 enhancers might similarly physically interact with their cognate promoters through a direct G4:G4 DNA based mechanism, we examined a second unrelated LG4 overlapping annotated enhancers on Chr12 (Figures 1E and 5A). Excitingly, as with the Chr5 LG4, after being incubated in G4-permissive conditions (+KCl), substantial, additive gel shifts were observed between ssDNAs corresponding to (i) the + strand of the EP400 promoter and the + strand of the Chr12 LG4 and (ii) the − strand of the EP400 promoter and the − strand of the Chr12 LG4 when folded together under G4 permissive conditions (Figure 5B, left). Further, additive gel shifts were also observed between ssDNAs corresponding to (i) the + strand of the ZNF84 promoter and the − strand of the Chr12 LG4 and (ii) the − strand of the ZNF84 promoter and the + strand of the Chr12 LG4 when folded together under G4 permissive conditions. Intriguingly, although less substantial, an additive gel shift was also observed between ssDNAs corresponding to the + strand of the ZNF84 promoter and the + strand of the Chr12 LG4 whereas no shift was observed between the − strand of the ZNF84 promoter and the − strand of the Chr12 LG4 (Figure 5B, middle, and Supplementary Figure S3). Perhaps most interestingly, however, although the GOLGA3 promoter (like the EP400 and ZNF84 promoters) is annotated by GeneHancer (Table 1) as likely interacting with the Chr12 LG4 enhancer, no interactions were observed between ssDNAs corresponding to either strand of the GOLGA3 promoter and ssDNAs corresponding to either strand of the Chr12 LG4 when folded together under G4 permissive conditions (Figure 5B, right).

### EMSA gel shifts involve G4 formation

**Figure 6.**
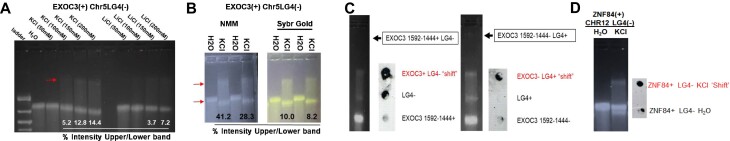
EMSA gel shifts involve G4 structures. (**A**) EMSA examining gel shifts formed by folding the + strand of the EXOC3 promoter and the − strand of the Chr5 LG4 together in [50–200 mM] KCl and LiCl solutions; 1.5% agarose Tris-glycine gel ran at 4°C for 8 h at 75 V and then stained for 24 h with SYBR Gold. (**B**) EMSA examining gel shifts formed by folding the + strand of the EXOC3 promoter and the − strand of the Chr5 LG4 together. Gels were stained with SYBR Gold to detect total DNA and NMM to detect G4 structures. (**C**) Dot blot of DNA isolated from the corresponding EMSA bands probed with the G4-specific antibody BG4. Dot blots of excised DNA are shown immediately to the right of their corresponding EMSA bands. Dot blot images are representative of triplicate experiments. (**D**) BG4 Dot blot using EMSA-isolated DNA corresponding to ZNF84 + Chr12LG4- folded in either H_2_O (left well) or KCl (top band in right lane). Dot blots of excised DNA are shown to the right of their corresponding EMSA bands.

To determine whether additive gel shifts involving specific promoters and LG4 enhancers are mediated by G4-based interactions, we employed three distinct strategies: (i) We evaluated the effects of increasing concentrations of potassium (and lithium) ions on EMSA additive gel shift formation. Importantly, we found a substantial, additive gel shift was clearly detected between ssDNAs corresponding to the + strand of the EXOC3 promoter and the − strand of the Chr5 LG4 when folded together in a [100 mM] KCl solution whereas no gel shift was observed when folded in a [100 mM] LiCl solution (Figure 6A). (ii) As another means of verifying that the bands corresponding to additive gel shifts were enriched for intact G4 structures, we sequentially stained EMSA gels containing ssDNAs corresponding to the + strand of the EXOC3 promoter and the − strand of the Chr5 LG4 with N-methyl mesoporphyrin IX (NMM), a G4-associating fluorescent dye (42), then with general DNA-associating SYBR Gold. Further supporting an enrichment for G4 structures in bands corresponding to additive gel shifts, we found NMM staining of additive gel shifts was ∼3.8-times more intense than SYBR Gold staining (Figure 6B). Finally, in addition to these analyses, we also (iii) physically excised ssDNA EMSA bands corresponding to the EXOC3 ‘−1592 to −1444’ promoter construct (to allow separation from the LG4), the Chr5 LG4, and the additive gel shift observed when these two ssDNAs are folded together in order to directly assess their G4 content via G4-specific dot blotting using the BG4 antibody. Notably, we found that while individual bands corresponding to the + strand of the EXOC3 promoter and the − strand of the Chr5 LG4 were both somewhat positive for BG4 staining, BG4 staining of the additive gel shift band corresponding to their interaction was substantially more robust. Even more pronounced, whereas we found little to no BG4 staining of individual bands corresponding to the -strand of the EXOC3 promoter or the + strand of the Chr5 LG4, in contrast, BG4 staining of the additive gel shift band corresponding to their interaction was markedly substantive (Figure 6C).

Similarly, the putatively complexed + strand of ZNF84 with Chr12 LG4- resulting from folding in KCl (denoted as ZNF84 + LG4- KCl shift) demonstrated enhanced BG4 staining compared to folding reactions of this DNA in H_2_O (Figure 6D). Collectively, these dot blots support the notion that the BG4 antibody, a G4-structure specific antibody, exhibits greater binding activity to promoter ssDNA when putatively complexed with LG4 ssDNA in G4 permissive conditions compared to non-complexed isoforms in either G4 permissive or prohibitive conditions (Figure 6C and D).

### Chr5 LG4 availability increases EXOC3 promoter activity in cells

**Figure 7.**
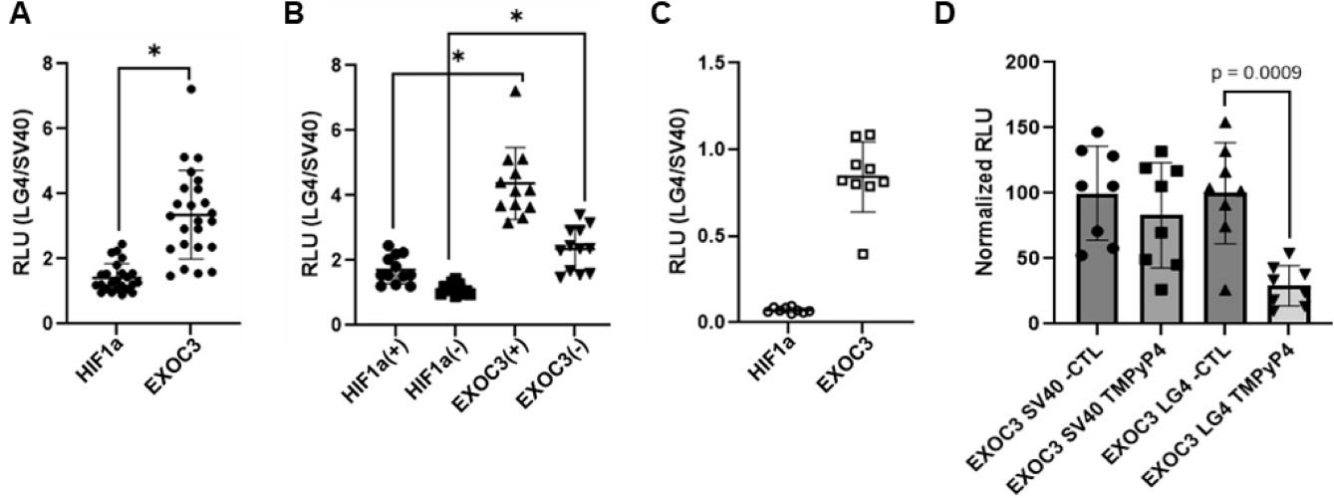
Chr5 LG4 presence increases EXOC3 promoter activity in cultured cells. Luminescence was quantified and normalized to control reporter cotransfections. Resulting ratios were plotted using GraphPad Prism software. Unpaired two-tailed *t*-test with Welsh’s correction was performed to determine significance. (**A**) Chr5 LG4 presence increases EXOC3 promoter activity. A549 cells were cotransfected with 50 ng of reporter plasmid (EXOC3 or HIF1a) and 50 ng of enhancer containing plasmid (Chr5 LG4 or SV40 in both orientations). (**B**) Luciferase assay stratified by enhancer orientation. Stratification of data displayed in (**A**) based on the orientation of the enhancer in TOPO pCR2.1. Transfection with sense LG4 was normalized to average RLU of transfections with sense SV40 and transfections with antisense LG4 were normalized to the average RLU of transfections with antisense SV40. *P < 0.0001. (**C**) A549 cells were transfected with 50 ng of reporter plasmid (pGL4-EXOC3-LG4, pGL4-HIF1a-LG4, pGL4-EXOC3-SV40 or pGL4-HIF1a-SV40) and LG4 RLU normalized to SV40 RLU. Resulting ratios were plotted using GraphPad Prism software. (**D**) A549 cells were transfected with 50 ng of reporter plasmid (pGL4-EXOC3-LG4 or pGL4-EXOC3-SV40) +/− TMPyP4 [100 nM] and RLU normalized to negative control (*n* = 8). Unpaired two-tailed *t*-test with welsh’s correction was performed to determine significance. Illustrations of reporter constructs are depicted in Supplementary Figure S5.

To confirm the Chr5 LG4 enhancer and EXOC3 promoter can interact in living cells, A549s were cotransfected with (i) a luciferase reporter driven by either the EXOC3 promoter or HIF1a control promoter and (ii) an enhancer containing plasmid (TOPO-Chr5 LG4 or TOPO-SV40 control enhancer). Transfections were performed with plasmids containing the enhancers in both orientations. Luminescence was measured at 24 h post-transfection and quantified as RLU. Experiments were repeated on three separate occasions to confirm reproducibility and afford sufficient replicates to establish significance (*n* = 24; *n* = 12 per enhancer orientation). RLU values obtained from transfections with promoter constructs and TOPO-Chr5 LG4 (+ or −) were normalized to the average RLU acquired from transfections with matched promoter reporter constructs and TOPO-SV40 (+ or −). Notably, the mean RLU ratio (LG4/SV40) observed for EXOC3 is nearly 2-fold higher than that of HIF1a (1.943 ± 0.2926, *P* < 0.0001) (Figure 7A). Moreover, stratification of this dataset based on enhancer orientation demonstrates that the Chr5 LG4 enhancer was able to significantly increase EXOC3 promoter activity regardless of orientation (*P* < 0.0001) (Figure 7B). In addition, we also find the Chr5 LG4 can similarly increase the expression of EXOC3, but not HIF1a, when located in *cis* (Figure 7C) (Supplementary Figure S5). Moreover, we find the ability of the Chr5 LG4 enhancer to activate the EXOC3 promoter in culture is significantly impeded by cellular treatment with the established G4 intercalating agent TMPyP4 (*P* = 0.0009) (Figure 7D), further supporting the claim that the interaction between the EXOC3 promoter and the Chr5 LG4 involves G4 structure formation (Figure 6).

## Discussion

Long G4-capable (LG4) loci are defined as genomic regions densely populated with minimal G4 capable sequences (GGGnGGGnGGGnGGG). Our previous work demonstrated that LG4s can assume higher order structures (23) although the requirements for, and determinants of, their assembly have not been fully elucidated. Moreover, although 217 of 301 previously identified LG4s overlap annotated enhancer elements, no direct participation of G4 structures within LG4s with regard to gene regulation have been reported to date. That said, despite the current generally accepted model for enhancer:promoter communication involving specificity of interaction being dictated by activator proteins independently bound to each regulatory element (1,11), this study was designed to test an alternative hypothesis: that LG4 enhancers physically interact with their cognate promoters via a direct G4:G4 DNA based mechanism.

Numerous studies have suggested potential roles for G4 DNA in enhancer activity to date. As examples: (i) G4 ChIP-Seq has repeatedly shown G4 structures are enriched in promoters and enhancers, (ii) G4 formation is significantly correlated with elevated transcriptional activity (43,44), (iii) independent genome-wide mappings of G4 structures in 2016 (43) and in 2022 found G4s located in enhancers are preferentially formed by inter-strand G4 folding (e.g. composite G4s containing only two GGG repeats from one strand of an enhancer) (45), and (iv) the formation of long-range intermolecular G4s being mediated by transcriptional regulatory proteins (that can selectively distinguish between inter vs intra molecular G4s) was also recently reported (46,47). Notably, a model for G4-based enhancer:promoter interaction was first proposed in 2015 based on the identification of a marked propensity for single enhancer:promoter pairs to contain potentially interacting minimal G4 motif components (15). In 2019, Hou *et al.* proposed a similar model for G4-based enhancer:promoter interactions after finding (i) >99% of G4s overlap known transcription factor binding sites (TFBSs), (ii) that G4s are significantly enriched at boundaries of TADs, and (iii) that frequent interactions occur between G4-containing regulatory elements (48).

That said, the current paradigm for how enhancer:promoter specificity is mediated involves the genome being divided into a hierarchical series of domains with enhancers regulating target promoters only within their own individual domains (49). At the top level, chromosomes occupy distinct spaces in the nucleus deemed chromosome territories (50) which can be divided into ∼1 Mbp regions known as TADs (51). TADs are themselves comprised of a series of smaller DNA loops known as insulated neighborhoods (INs) formed by interactions between two DNA sites bound by CTCF and the cohesion complex and typically contain 1–8 genes and 1 or 2 enhancers (52) (similar to the regulatory neighborhoods proposed in Figure 1D and E). Enhancers located within TADs are believed to only interact with genes located in their respective neighborhood (53) as when TAD boundaries are disrupted, aberrant enhancer:promoter interactions result in significant gene misexpressions (54–56). Although G4 DNA was only recently directly linked to CTCF recruitment (57), roles for CTCF and cohesin in 3D genomic organization (58) and for CTCF promoter binding as a mediator of long distance enhancer dependent transcription (28) have long been established. Of note, Hou *et al.* found that G4s are significantly enriched at TAD boundaries and that G4 content strongly correlates with occupancy of architectural proteins critical for TAD formation. They found that (i) adjacent, G4-containing boundaries frequently interact, (ii) the insulation abilities of CTCF binding sites and TAD boundaries are significantly reinforced by G4s, (iii) that >99% of G4s at these positions overlap TFBSs, and (iv) that CTCF and cohesin binding sites are preferentially located near G4s. Collectively, these findings clearly support G4 involvement in loop extrusion and distal interactions between enhancers and promoters (48) [reminiscent of local chromosomal compartment formation being directed by Rif1 protein composite G4 binding in fission yeast (59)].

Similar to Hou *et al.*, we previously identified and reported significant associations (*P* < 0.0001) between our LG4 loci and ChIP-seq peaks corresponding to 26 different TFs including CTCF, YY1 and SP1 (23) [known to associate with promoter G4s (60)]. YY1 has also been shown to contribute to enhancer:promoter interactions (61), and in 2021, Li *et al.*identified G4 binding as a molecular requirement for YY1-driven long-range enhancer:promoter interactions (62). Notably, they found displacement of YY1 from G4 structures with small-molecule G4 ligands, unwinding of G4 structures by overexpression of BLM helicase, and CRISPR–Cas9 mutation of G4-forming promoter sequences could each disrupt YY1-mediated DNA looping. In addition, they also showed expression of genes harboring G4 structures in their promoters could be significantly perturbed by either inhibiting YY1:G4 binding (by the use of G4-stabilizing ligands PDS or TMPyP4) or depletion of YY1 by RNA interference (62).

Against this background, despite a mounting body of evidence implicating G4 involvement, the current, generally accepted model for enhancer:promoter communication posits that enhancer:promoter specificity is dictated by interactions between activator proteins independently bound to each (1,11). The work detailed in this report, however, suggests that this may not be the only mechanism involved with governing these critical interactions. While this study was designed to test the hypothesis that LG4 enhancers physically interact with their cognate promoters via a direct G4:G4 DNA based mechanism, we feel it is critical to note that the aforementioned (and many additional) characterized roles for DNA-binding proteins in bringing enhancers and promoters more proximal to one another are in no way incompatible with direct G4 interaction and are almost certainly involved with and critical for mediating the interactions described in our work. While it is clear that enhancers regulate distal genes by genomic looping and physical interaction, how enhancers target the right genes remains less certain. Enhancers frequently demonstrate selectivity for specific promoters within individual TADs with enhancers routinely crossing other intervening genes that are not activated by the enhancer during the ‘search’ for a target promoter (2,10,11). Several models have been proposed to explain promoter selectivity, but to date, the mechanisms responsible for the majority of specific enhancer:promoter interactions remain unresolved. That said, beyond simply establishing that LG4s and their putative target promoters are proximally located, this study provides experimental evidence supporting direct, specific interactions between ssDNAs corresponding to (i) a LG4 found on human Chromosome 5 (Chr5 LG4) and two of its predicted target promoters (Figure 3A) and (ii) a LG4 found on human Chromosome 12 (Chr12 LG4) and two of its predicted target promoters but (perhaps most importantly) not the more proximally located, intervening GOLGA3 promoter (Figure 5A). As such, the work presented in this study clearly suggests a direct role for enhancer and promoter G4 DNA sequence interaction in mediating enhancer:promoter specificity.

Further of note, we find a 35 bp portion of the EXOC3 promoter is required for and independently capable of interacting with the Chr5 LG4 (Figure 5), and interestingly, that this 35 bp portion is devoid of any minimal G4 capable sequence motifs (Figure 4E and Supplementary Figure S4). As such, we suggest the inability of this region to form independent G4 structures coupled with the observation that G4 formation is required for this region to engage in DNA-based enhancer:promoter interaction (Figure 6) is clearly in agreement with the general model proposed in the ‘Graphical Hypothesis’ in which LG4 enhancers physically interact with gene promoters by forming composite G4 structures where both the LG4 and cognate promoter contribute a portion of the necessary sequence for G4 formation.

Data presented in this study also suggests that individual LG4 enhancers regulate a specific set of target promoters potentially coordinating their expressions. We find the average number of G triplets (GGG) contributing to composite G4 formation within a single LG4 is 74-times the number of available G triplets found in the average inferred target-gene promoter (23), leading us to speculate that the high number of available G4 donor sequences within a single LG4 may allow LG4 enhancers to act as long ‘Velcro-like’ regions that simultaneously interact with a number of neighboring gene promoters coordinating their expressions (see ‘Graphical Hypothesis’). Although conclusively establishing a role for LG4s in promoter coordination is beyond the scope of the current study and will ultimately require more extensive examination, we find several lines of evidence suggesting that the expressions of genes regulated by LG4 enhancers are likely coordinated. Particularly of note, our preliminary assessment of TCGA patient expression data (63) finds the expressions of CEP72, BRD9, and PDCD6 to be significantly, positively correlated with EXOC3 expression, and similarly, the expressions of DDX51, POLE and ZNF84 to be significantly, positively correlated with EP400 expression in lung adenocarcinoma (Figure 1D and E, and Supplementary Figure S6).

Regardless of the ability to coordinate gene expression, we suggest the regulatory action which LG4s impose on their respective target genes is likely disease relevant. Particularly of note, in 2020, a genome-wide association study designed to identify genomic modifiers of cystic fibrosis (CF) identified 28 genes (near known CF-associated loci) whose expressions strongly correlate with CF lung disease severity in patients (64). Strikingly, 43% (12 of 28) of these disease modifying genes (e.g. BRD9, CEP72, EXOC3, TPPP, ZDHHC11) reside in the Chr5 LG4 neighborhood depicted in Figure 3A, and what’s more, the expressions of CEP72, EXOC3, TPPP and ZDHHC11 were identified as the four most highly associated with CF severity genome wide. In addition, this study also found a marked number of SNPs correlated with altered EXOC3, CEP72 and TPPP expressions were not located within the respective promoters of these genes but instead within an ∼40 kb window roughly centered around the Chr5 LG4 (64). More recently, a subsequent genomic and transcriptomic association study of 7840 CF patients (35) similarly identified four CF-relevant SNPs (located within the Chr5 LG4) significantly associated with the expressions of EXOC3 and CEP72 (Supplementary Table S3). Strikingly, these SNPs showed significant eQTLs with the expression of these genes by GTEx in multiple human tissues including lung (36,64–69). Although the associations between these SNPs and CF severity are purely correlative for now, it is tempting to speculate that perturbation of the central LG4 enhancer regulating these genes might have substantive consequences on CF lung disease phenotype. That said, although we have not yet undertaken a comprehensive examination aimed at identifying similar potential associations between other diseases/genetic pathways and the 300 additional LG4s identified in our original study, we do plan to explore this in depth in the near future.

In addition to this, this report includes several lines of evidence indicating LG4s and their putative target promoters are proximally located in the nucleus. In one of these, we find EQuIP pulldowns using probes specific for Chr5 LG4 DNA to be highly enriched with DNA from the EXOC3 and CEP72 promoters (Figure 2B). Of note, although we have only employed EQuIP as one of several lines of evidence supporting LG4 enhancer:promoter proximity in the current study, our group is actively engaged with developing a strategy for sequencing EQuIP isolated DNA in order to comprehensively map the full range of promoters associating with individual LG4 enhancers. While comprehensively sequencing EQuIP isolated DNA has proven more technically challenging than originally anticipated, realizing ‘EQuIP-seq’ unquestionably constitutes another significant future direction for our work.

Further of note, although the current study provides strong evidence supporting a role for enhancer and promoter G4 DNA sequence interaction in mediating enhancer:promoter specificity, it also raises some critical new questions regarding how promoter:LG4 interactions are potentially regulated/function *in vivo*. For example, (i) what are the critical sequences required for mediating interaction and promoter specificity? In addition, the current study only biochemically interrogated two LG4s limiting our ability to confidently conclude that the regulatory mechanism described in this work represents a general mechanism of LG4 action. As such, (ii) how widespread are G4-based enhancer:promoter interactions? Further, while tissue specific enhancer regulation is well-established, the current study does not explore the cellular context required for LG4 interactions to occur. In light of this, (iii) is it possible for a LG4 enhancer to regulate one set of promoters in one cell type and another set of promoters, or to be inactive altogether, in other types? And finally, (iv) are LG4 enhancer:promoter interactions conserved across species, and if so, can this help define minimally interacting sequences?

In conclusion, in contrast to the generally accepted model for enhancer:promoter communication in which enhancer:promoter engagement is dictated by interactions between activator proteins, the work presented in this report describes a novel G4 DNA-based mechanism capable of mediating enhancer:promoter interaction *in vitro* and confirms LG4s and their target promoters are proximally located in the nucleus supporting the likely relevance of this mechanism *in vivo*. That said, while our work successfully (i) provided multiple lines of evidence indicating that specific LG4s and their target promoters are proximally located in the nucleus, (ii) biochemically demonstrated that ssDNAs corresponding to individual LG4s and promoters specifically associate with each other in a G4-dependent manner; and (iii) showed that the ability of the Chr5 LG4 enhancer to activate a target promoter in culture is significantly impeded by cellular treatment with an established G4 intercalating agent, TMPyP4; establishing clear roles for G4-mediated targeting in facilitating enhancer:promoter specificity *in vivo* will clearly require additional study. In addition to this, while this work provides the first ever experimental characterization of enhancer:promoter interactions facilitated by a direct G4:G4 DNA based mechanism, other functional roles for G4 DNA structures have long been established [e.g. intramolecular telomere DNA G4 formation preventing telomere extension by limiting access to telomerase (70)]. As such, whereas storing biological information is unquestionably the primary function of DNA, we suggest functional roles for ssDNA structures are likely more prevalent than currently appreciated and warrant further study.

## Supplementary Material

gkae1180_Supplemental_Files

## Data Availability

All data, including raw gel images, plasmid sequences and raw luciferase data, are available upon request.
