# Supplementary material for: Long G4-rich enhancers target promoters via a G4 DNA-based mechanism
Source: Nucleic Acids Res. 2024 Dec 10;53(2):gkae1180. doi: 10.1093/nar/gkae1180 (PMC11754661; doi:10.1093/nar/gkae1180)
Supplement: gkae1180_Supplemental_Files [file gkae1180_supplemental_files.zip › Combined Supplemental Figures and Tables.pdf]

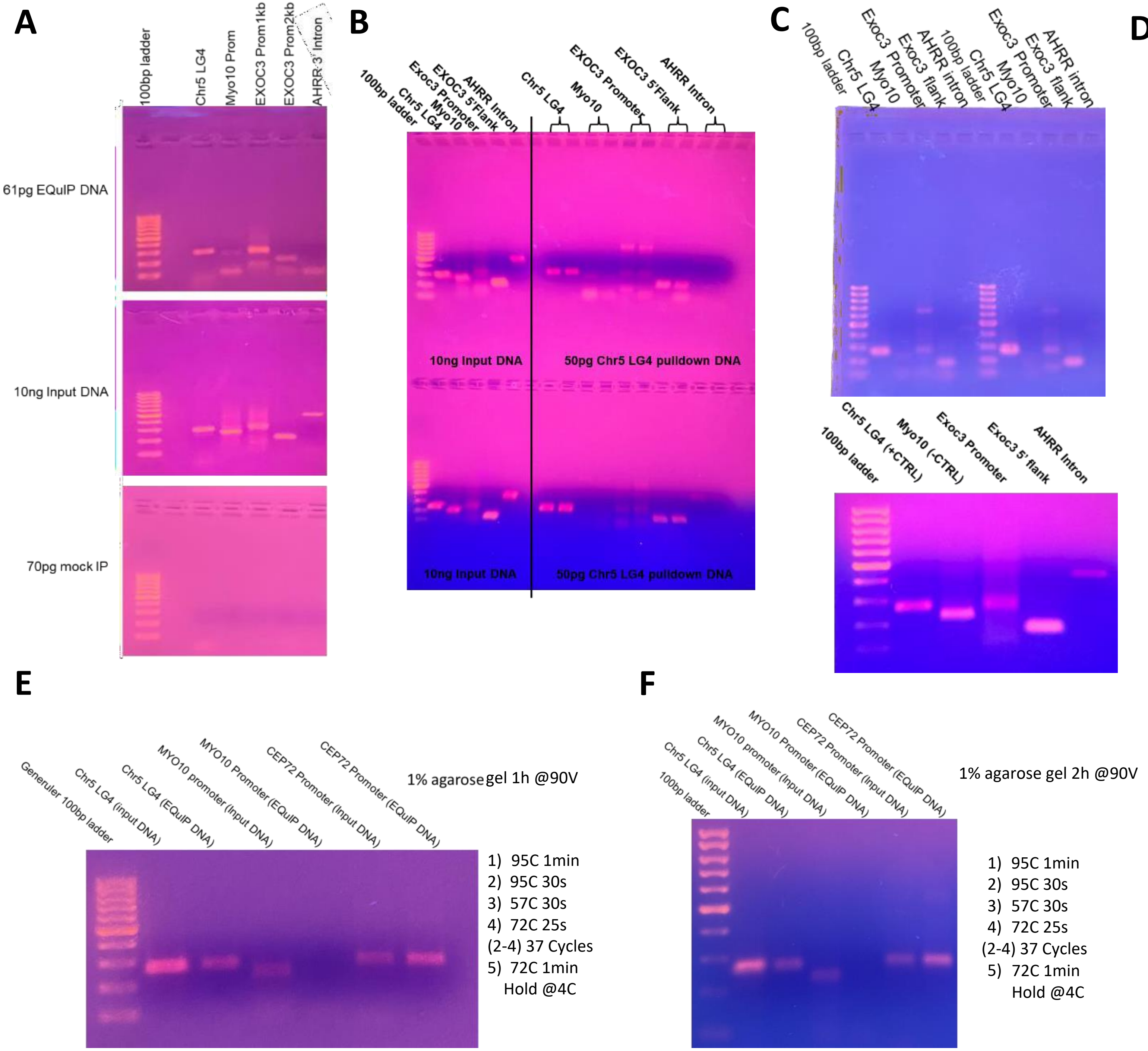

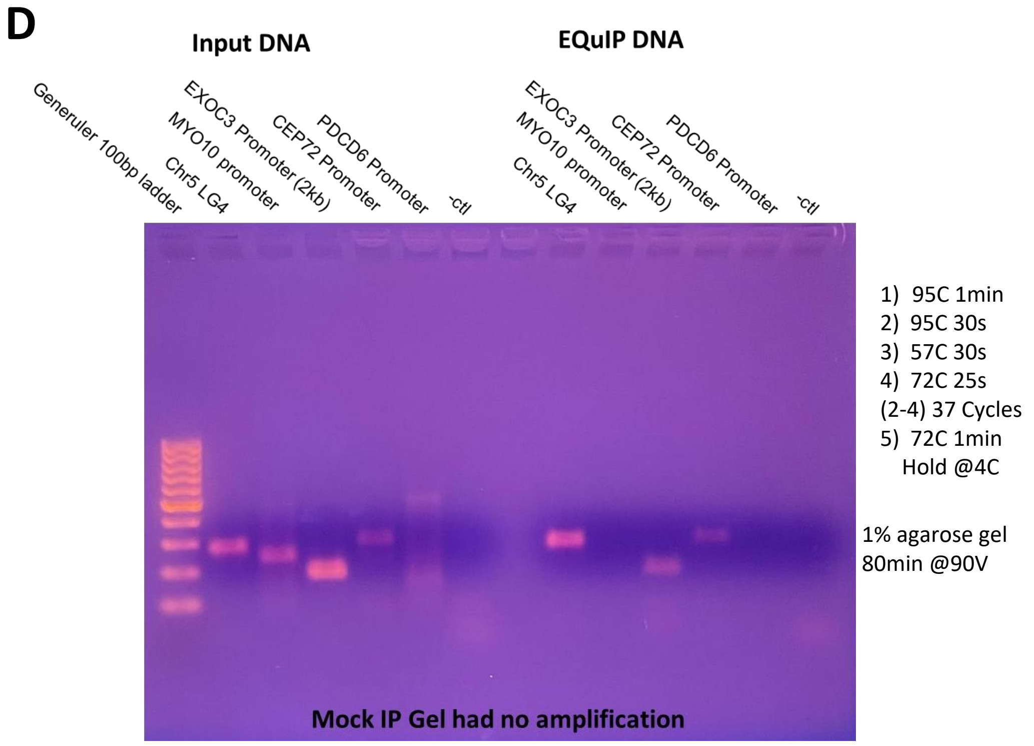

Supplemental Figure 1. EQuIP (Enhancer Quadruplex ImmunoPrecipitation) PCR full gels. (**A**) Full gels of indicated PCRs. Full gels of PCRs employing DNA template isolated from PC3 cell EQuIP pulldowns, IPs with nonspecific probes (scramIP) or total input DNA collected prior to IP. PCR amplicons (~300bp) located: (Chr5 LG4) within the Chr5 LG4, (Myo10 Prom) ~200 bp upstream of the transcription start site of the Myo10 protein coding gene, (EXOC3 Prom1kb) ~1.4 kbp upstream of the transcription start site of the EXOC3 protein coding gene, (EXOC3 Prom2kb, 5'Flank) ~2.0 kbp upstream of the transcription start site of the EXOC3 protein coding gene, (AHRR 3' Intron) within an intron located near the 3' end of the AHRR protein coding gene. PCR amplicons were verified by sequencing. (**B**) Full gels of PCRs (as named in A) run in duplicate employing input and LG4 pulldowns as template. Two sets of IPs are shown (one set loaded in the upper wells and another in the lower wells) that were isolated independently on separate occasions. (**C**) (Top) Full gel of PCRs (as named in A) run in duplicate (one shown on the left and another on the right) employing LG4 pulldowns as template. (Bottom) Full gel of PCRs employing input DNA (from the same experiment) as template although input PCRs were not run in duplicate. The LG4 pulldown shown was isolated independently on a separate occasion from the IPs shown in **A** and **B**. PCRs run identically to those in **A** and **B** excepting PCR was run for 32 cycles. (**D**) Full gels of PCRs shown in **Figure 2B**. (**E,F**) Full gels of PCRs (as named in D) run in duplicate employing input and LG4 pulldowns as template.

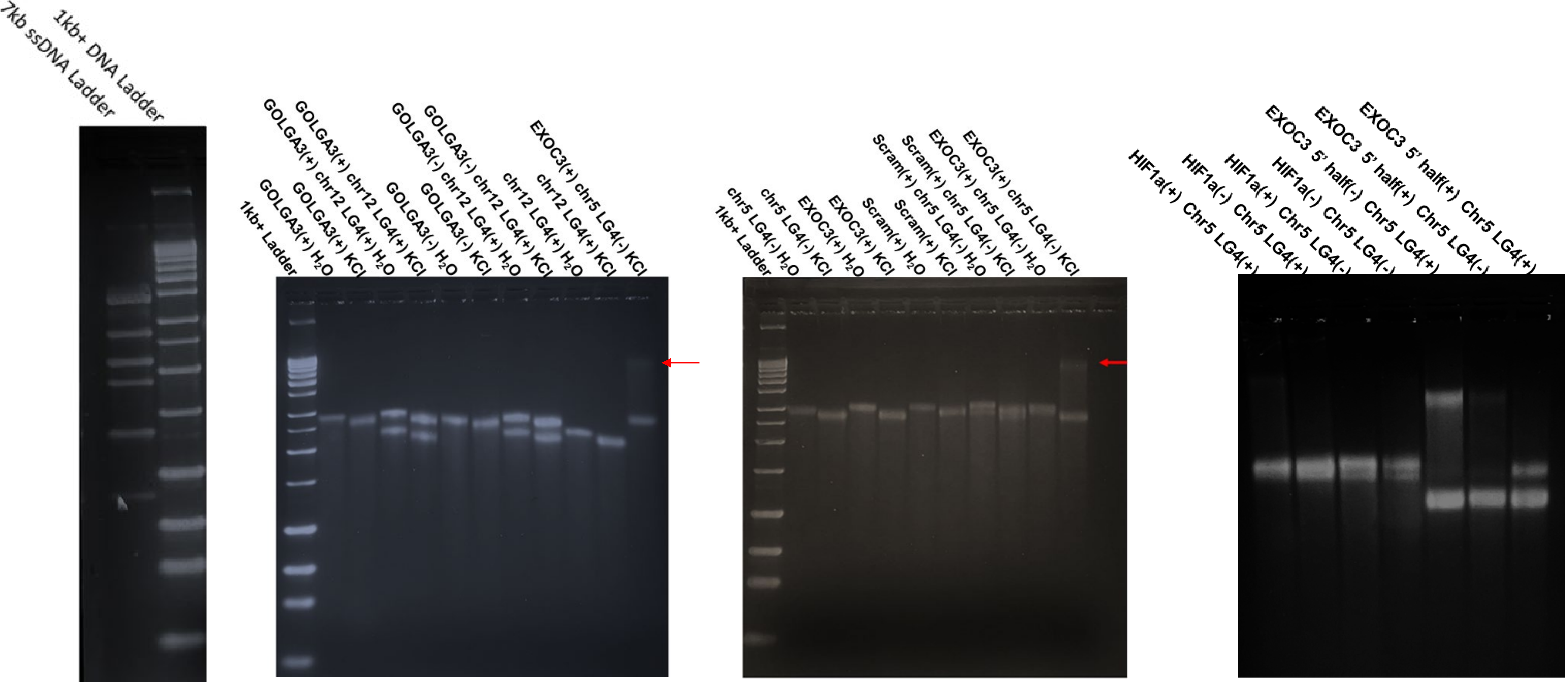

Supplemental Figure 2. Relative sizes of ssDNA sequences and comparing shift intensities . Images of 1.5% agarose Tris-glycine gels ran at 4°C for 8 h at 75 V and then stained for 24 h with SYBR GOLD. 7kb ssDNA Ladder (Revvity cat# CLS157950) has 6 size standards at 1100 nt, 2100 nt, 3200 nt, 4000 nt, 5100 nt and 7200 nt. Each construct was run on a gel in either the unfolded (H2O) or folded (KCl) state as indicated above the image. The red arrow denotes the gel shift observed when the EXOC3 promoter positive strand and LG4 negative strand are folded together. Scram: EXOC3-size-matched, scrambled control. Samples including more than one construct were folded together. The samples displayed in the gel image on the right were each folded in the presence of KCl to directly compare gel shift intensities resulting from folding Chr5 LG4 and EXOC3 5' half constructs with nonspecific smearing observed from folding reactions of Chr5 LG4 and HIF1a.

Supplemental Figure 3. EMSA quantifications. Quantifications of the EMSAs displayed in figures 3C, 4B, 4D, 4F, and 5B were performed using the ImageJ gel analysis function. Lanes were plotted and relative band intensity was determined by calculating the area of the corresponding peak whereby a larger peak area is indicative of greater band intensity at that position. The peaks are numbered according to the order in which they appear in the quantification table. The ssDNA within each well is listed below its corresponding plot.

Figure 3C

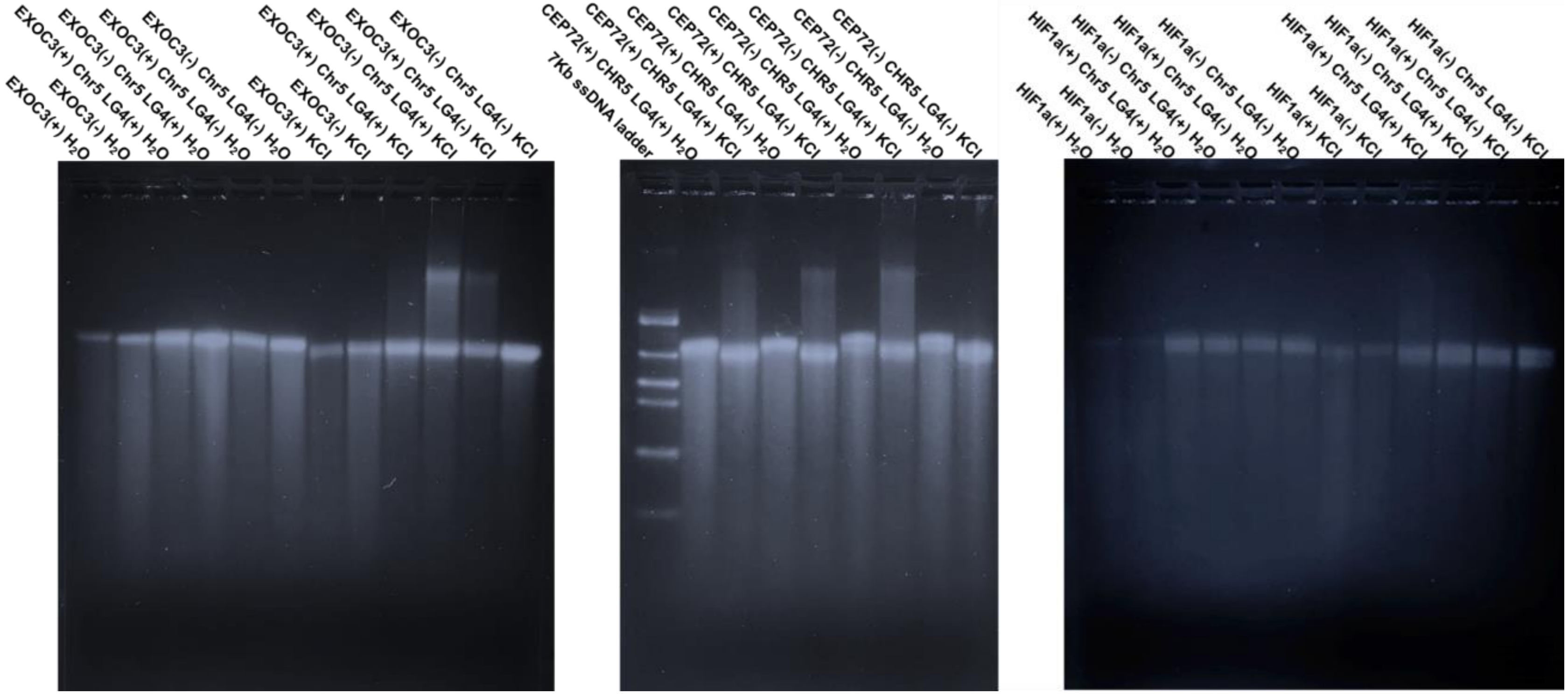

EXOC3(+) H_2O
EXOC3(+) Chr5 LG4(+) H_2O
EXOC3(-) H_2O
EXOC3(-) Chr5 LG4(+) H_2O
EXOC3(+) Chr5 LG4(-) H_2O
EXOC3(-) Chr5 LG4(-) H_2O
EXOC3(+) KCl
EXOC3(+) Chr5 LG4(+) KCl
EXOC3(-) KCl
EXOC3(-) Chr5 LG4(+) KCl
EXOC3(+) Chr5 LG4(-) KCl
EXOC3(-) Chr5 LG4(-) KCl
7Kb ssDNA ladder
CEP72(+) CHR5 LG4(+) H_2O
CEP72(+) CHR5 LG4(+) KCl
CEP72(+) CHR5 LG4(-) H_2O
CEP72(+) CHR5 LG4(-) KCl
CEP72(-) CHR5 LG4(+) H_2O
CEP72(-) CHR5 LG4(+) KCl
CEP72(-) CHR5 LG4(-) H_2O
CEP72(-) CHR5 LG4(-) KCl
HIF1a(+) H_2O
HIF1a(+) Chr5 LG4(+) H_2O
HIF1a(-) H_2O
HIF1a(-) Chr5 LG4(+) H_2O
HIF1a(+) Chr5 LG4(-) H_2O
HIF1a(-) Chr5 LG4(-) H_2O
HIF1a(+) KCl
HIF1a(+) Chr5 LG4(+) KCl
HIF1a(-) KCl
HIF1a(-) Chr5 LG4(+) KCl
HIF1a(+) Chr5 LG4(-) KCl
HIF1a(-) Chr5 LG4(-) KCl

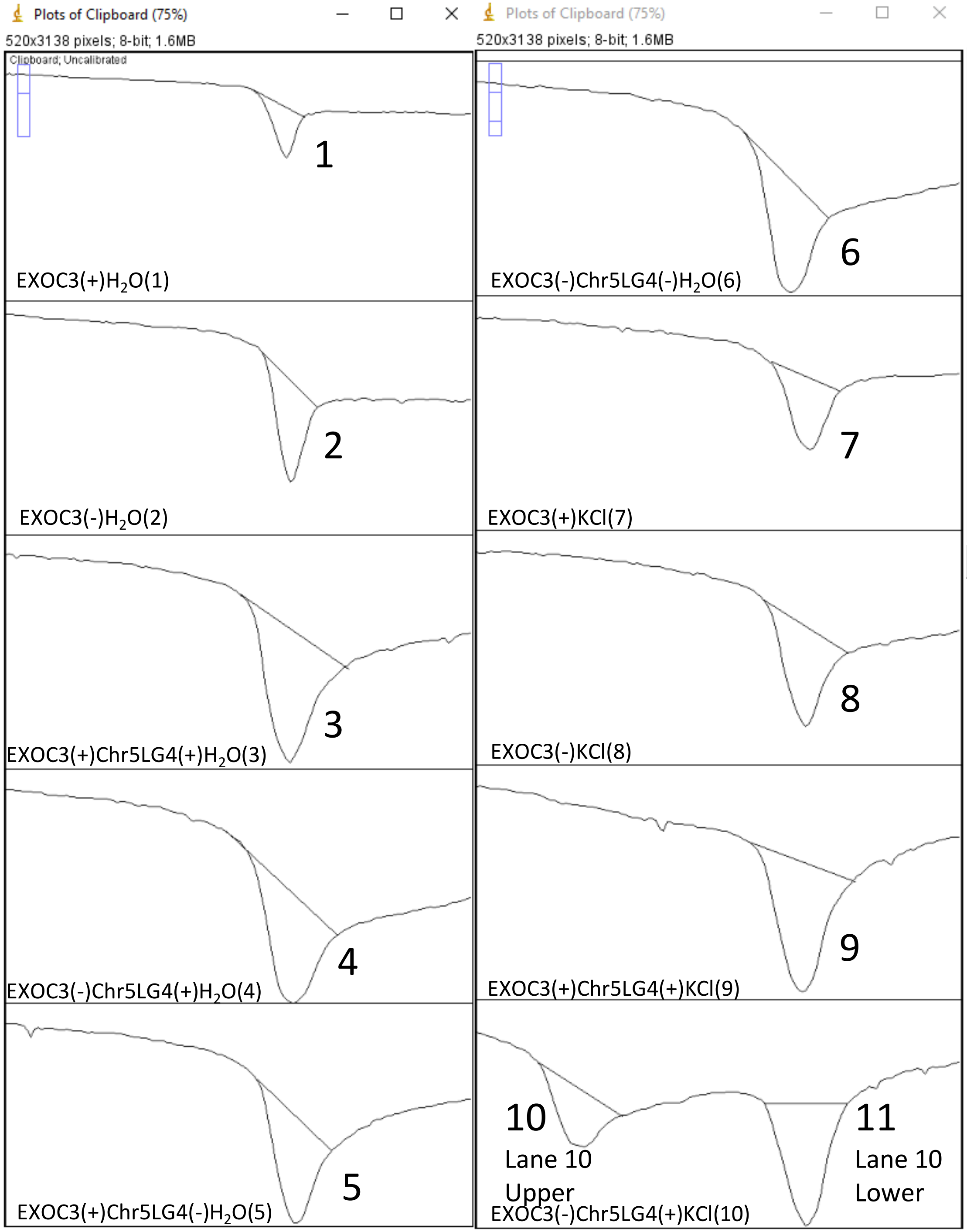

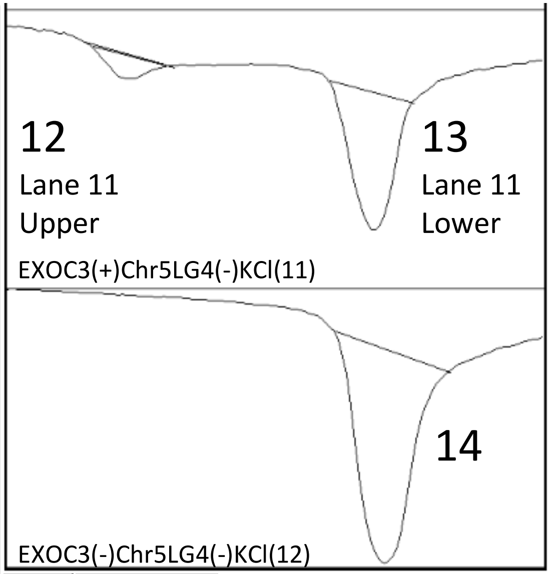

	Area
1	1416.154
2	3473.317
3	8126.693
4	6738.108
5	5314.894
6	5930.380
7	3219.539
8	4855.116
9	7650.530
10	3256.388
11	6312.518
12	920.012
13	5691.296
14	11149.823

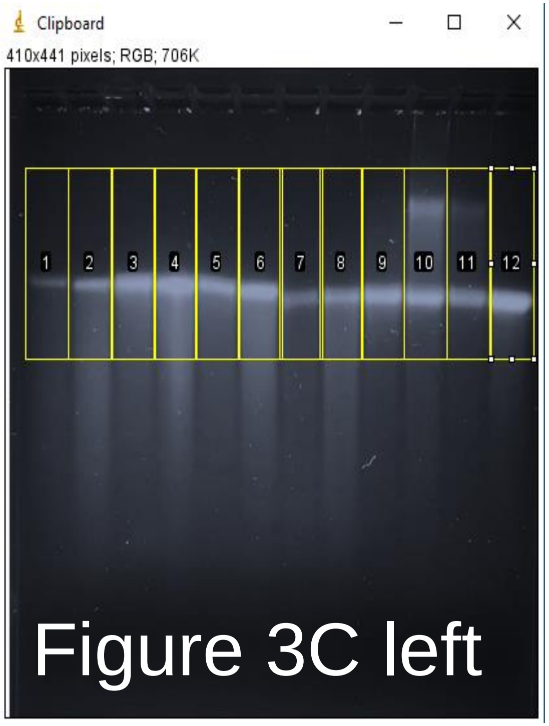

Figure 3C left

Plots of Clipboard (50%)

604x2434 pixels; 8-bit; 1.4MB

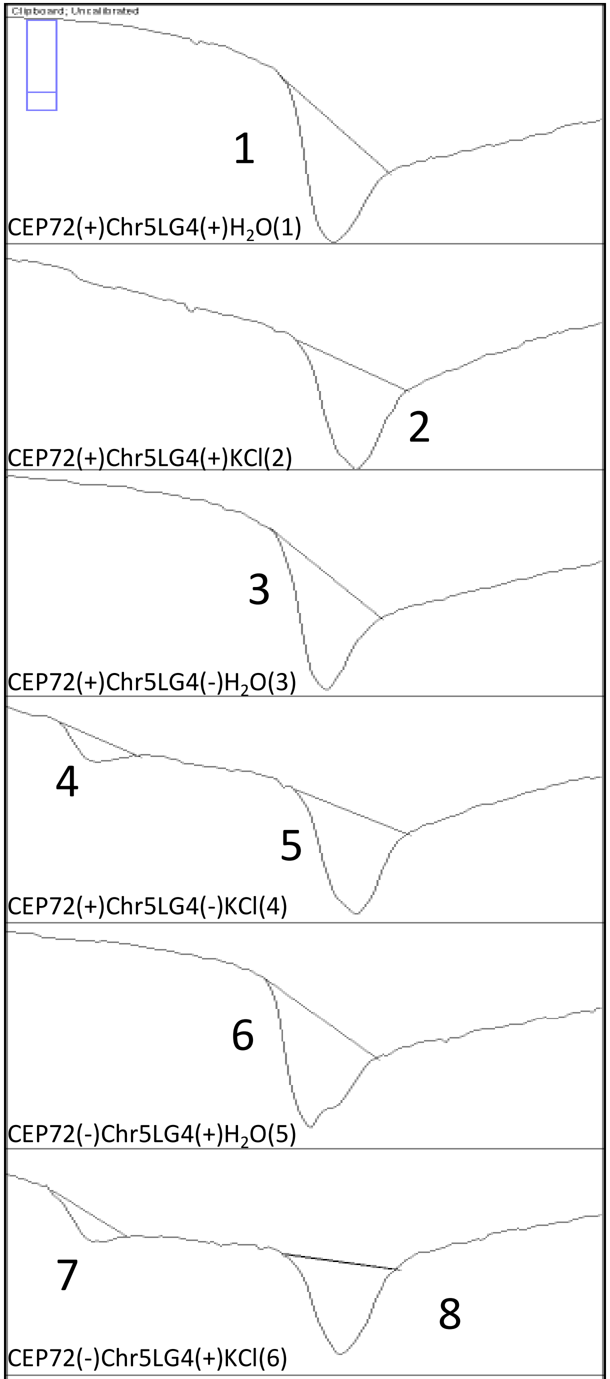

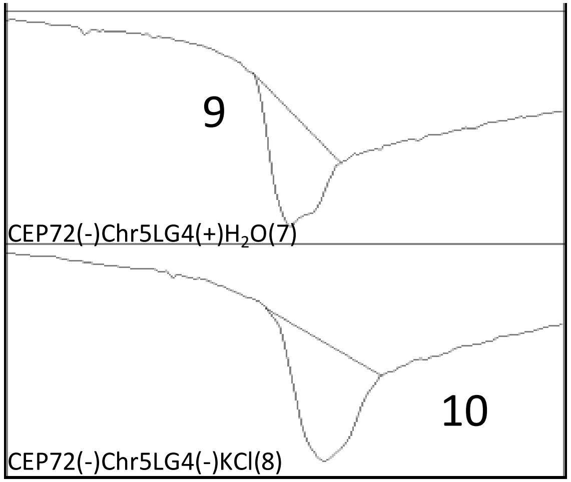

pboard

4 pixels; RGB; 548K

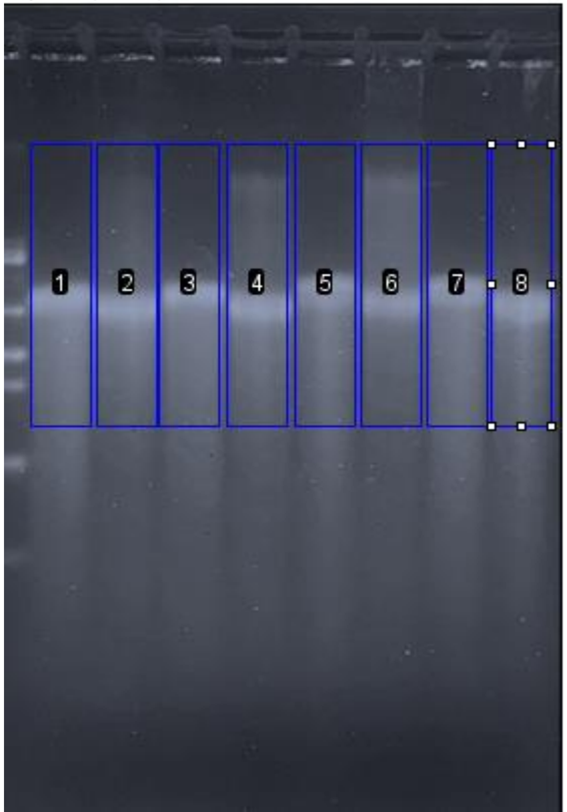

Results

File Edit Font

	Area
1	9291.421
2	8288.037
3	8653.785
4	1484.225
5	8251.794
6	9259.957
7	1504.903
8	7104.338
9	8042.037
10	10435.128

Figure 3C middle

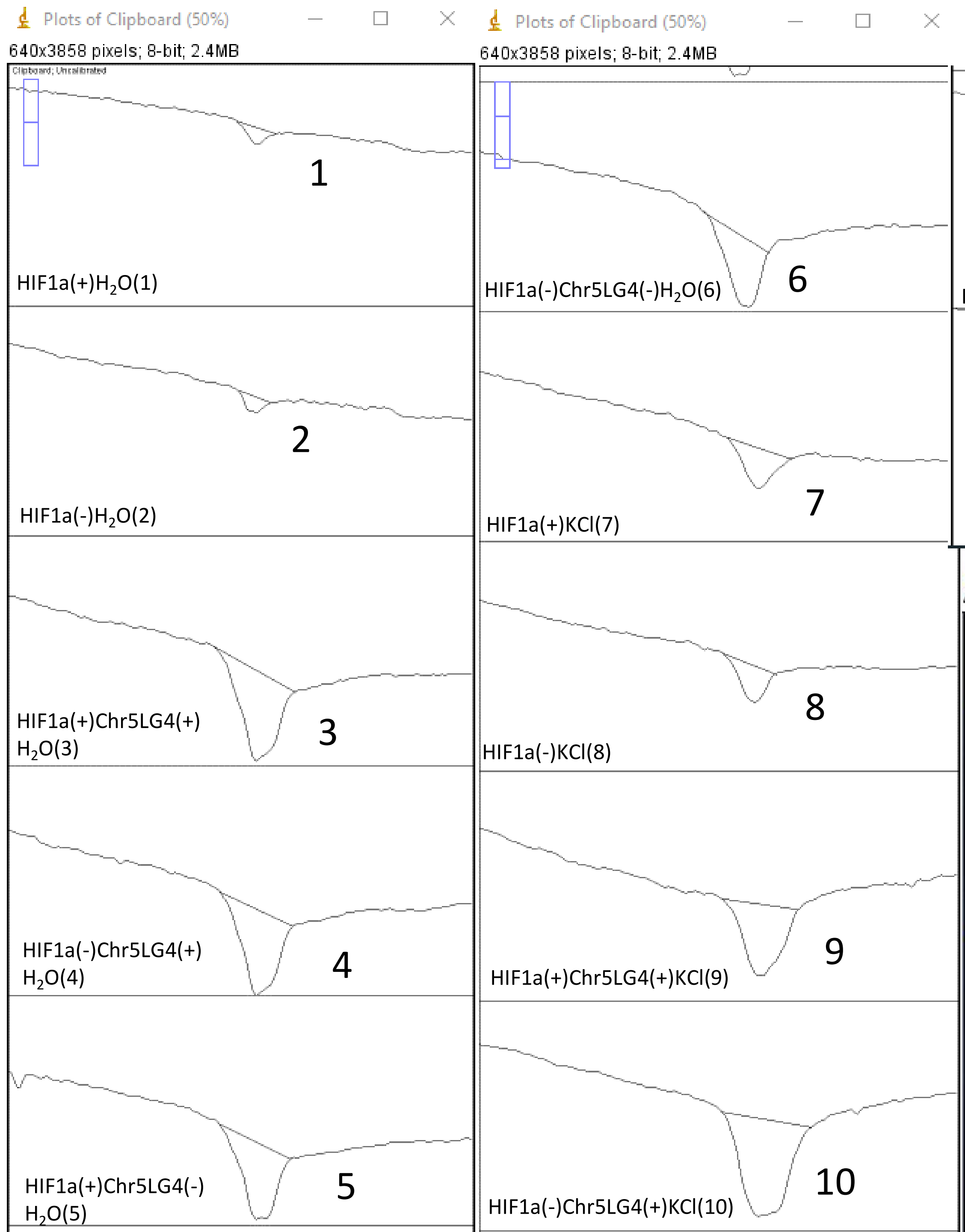

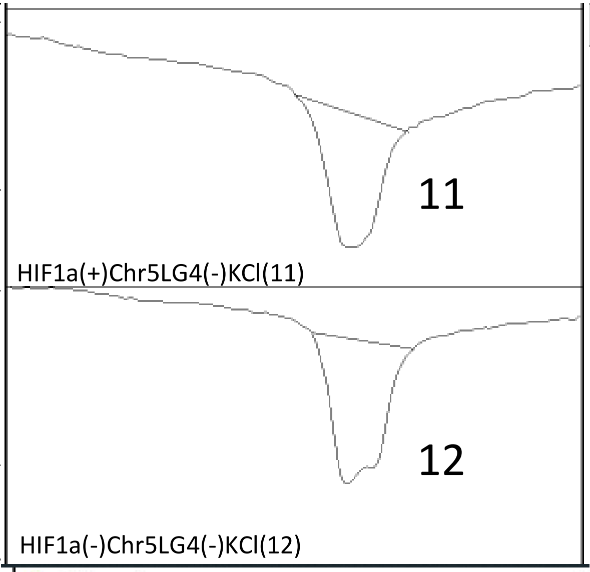

	Area
1	632.891
2	615.648
3	6791.258
4	6136.581
5	5422.459
6	4467.974
7	2449.953
8	1874.933
9	5539.246
10	9313.853
11	9889.359
12	10508.853

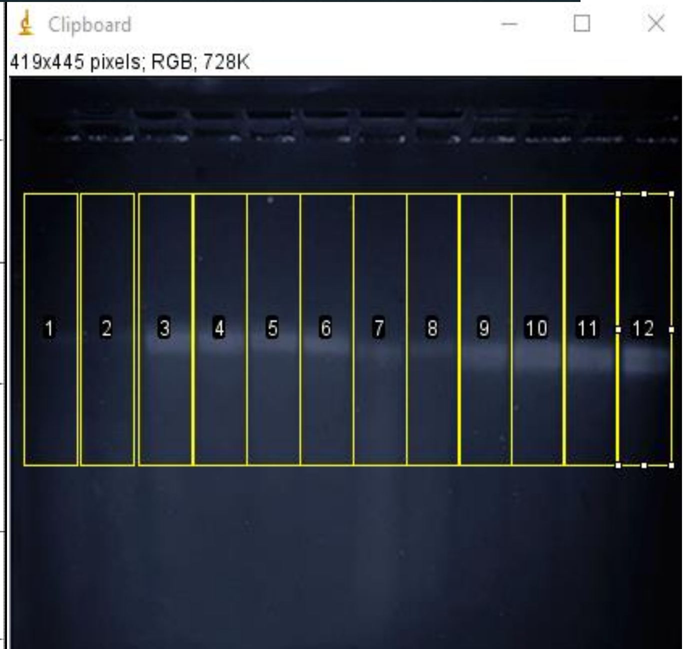

Figure 3C right

Figure 4A,B

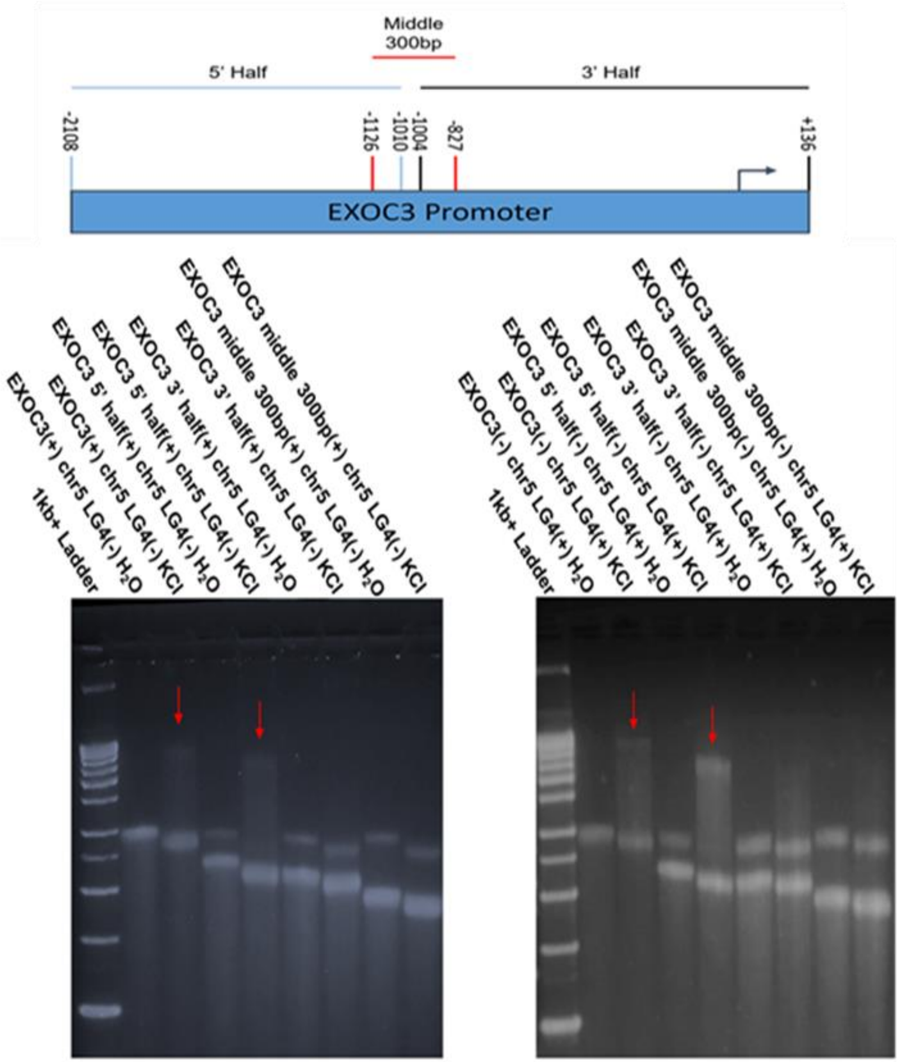
Middle
300bp
5' Half
3' Half
-2108
-1126
-1010
-1004
-827
+136
EXOC3 Promoter
1kb+ Ladder
EXOC3(+) chr5 LG4(-) H2O
EXOC3(+) chr5 LG4(-) KCl
EXOC3 5' half(+) chr5 LG4(-) H2O
EXOC3 5' half(+) chr5 LG4(-) KCl
EXOC3 3' half(+) chr5 LG4(-) H2O
EXOC3 3' half(+) chr5 LG4(-) KCl
EXOC3 middle 300bp(+) chr5 LG4(-) H2O
EXOC3 middle 300bp(+) chr5 LG4(-) KCl
1kb+ Ladder
EXOC3(-) chr5 LG4(+) H2O
EXOC3(-) chr5 LG4(+) KCl
EXOC3 5' half(-) chr5 LG4(+) H2O
EXOC3 5' half(-) chr5 LG4(+) KCl
EXOC3 3' half(-) chr5 LG4(+) H2O
EXOC3 3' half(-) chr5 LG4(+) KCl
EXOC3 middle 300bp(-) chr5 LG4(+) H2O
EXOC3 middle 300bp(-) chr5 LG4(+) KCl

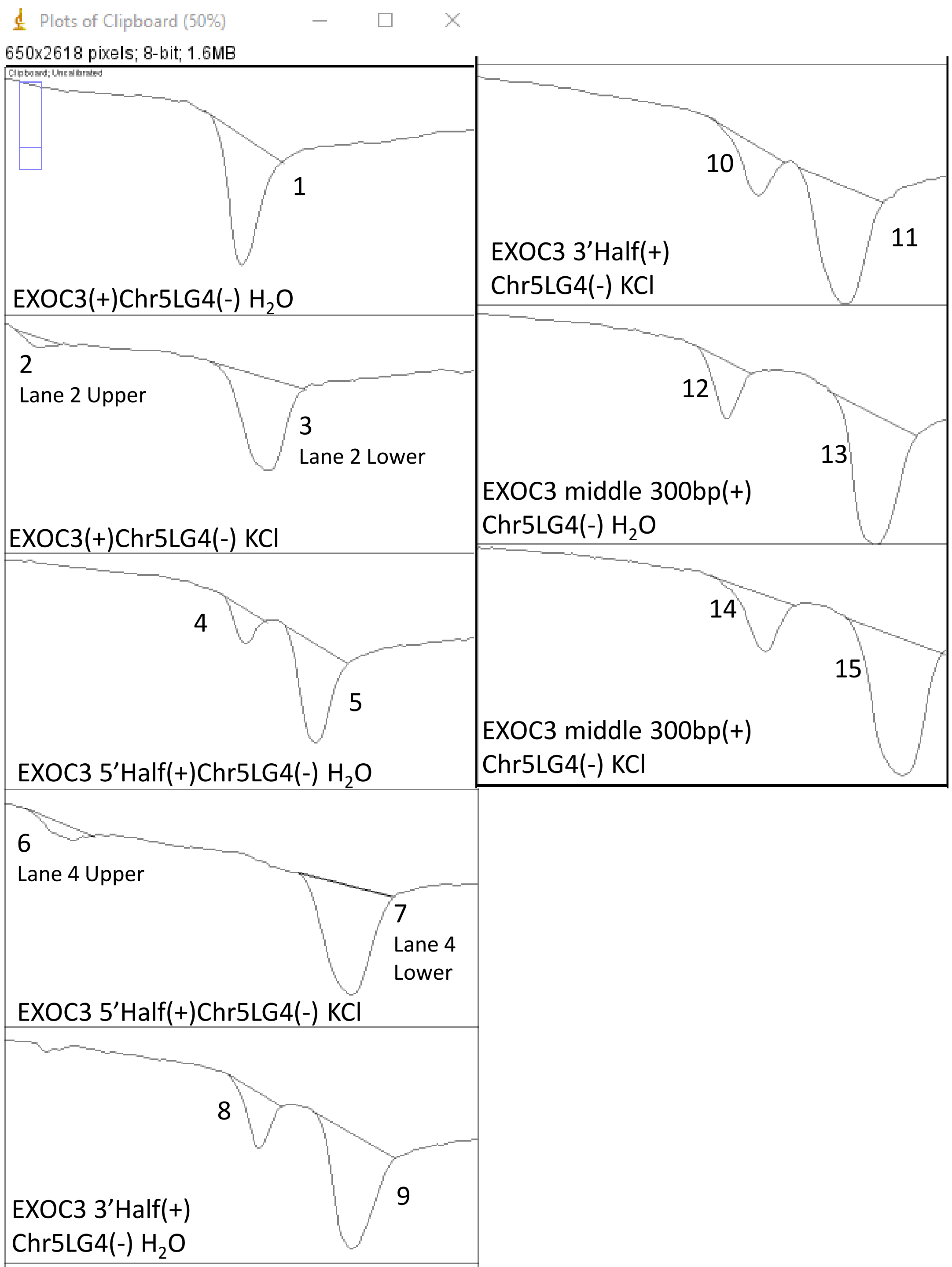

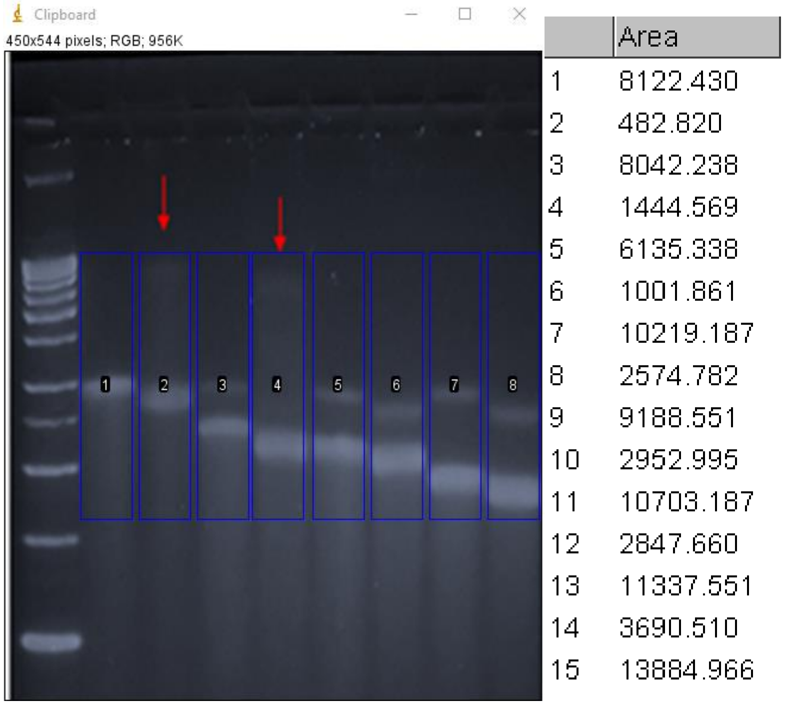

	Area
1	8122.430
2	482.820
3	8042.238
4	1444.569
5	6135.338
6	1001.861
7	10219.187
8	2574.782
9	9188.551
10	2952.995
11	10703.187
12	2847.660
13	11337.551
14	3690.510
15	13884.966

Figure 4B, left

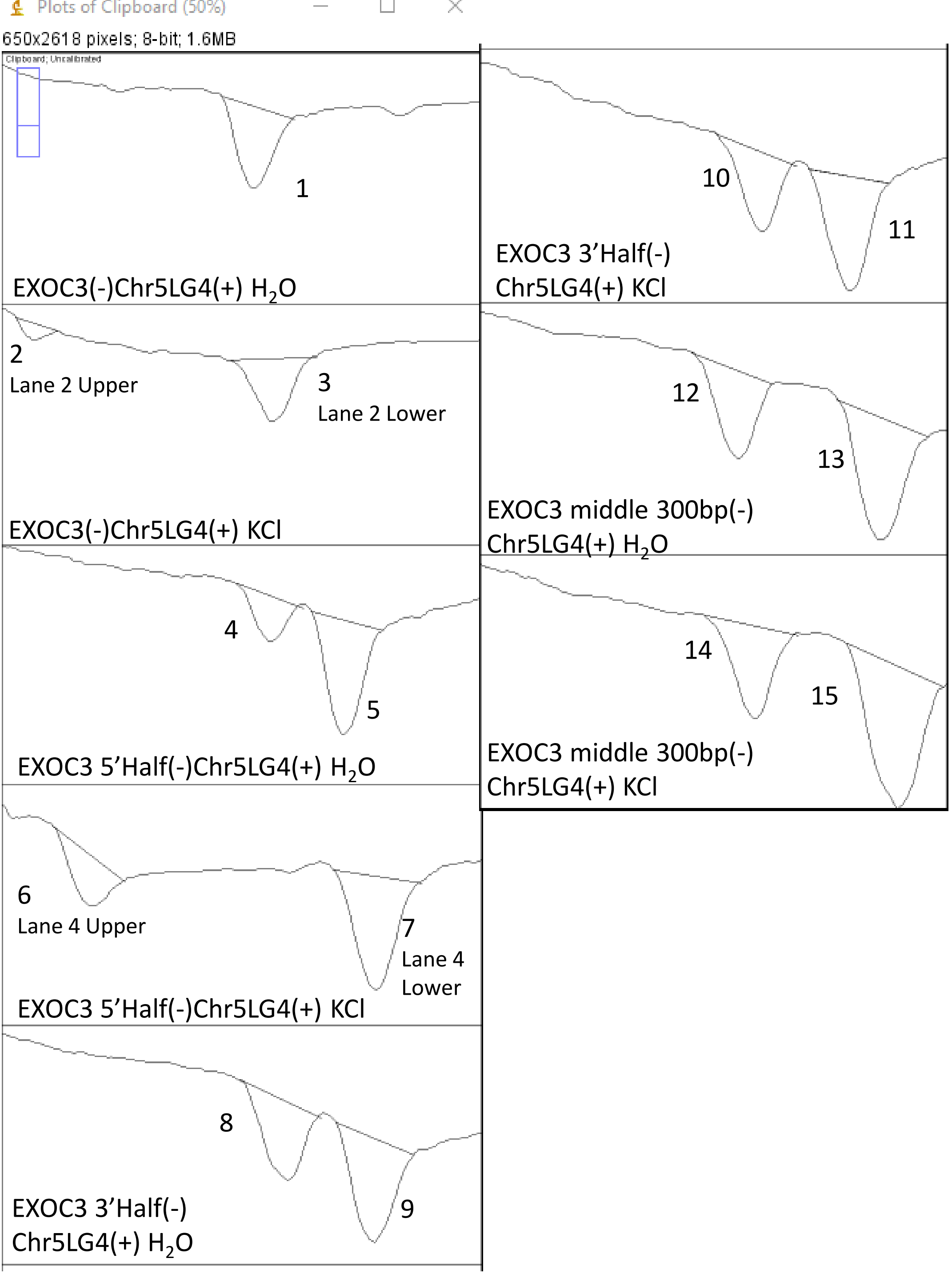

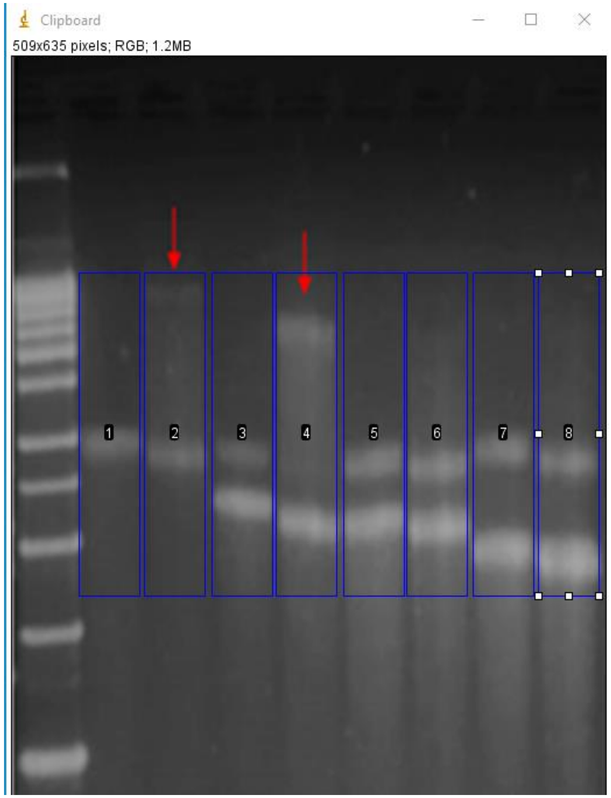

	Area
1	5798.853
2	757.355
3	4578.125
4	2791.246
5	7914.731
6	3759.530
7	9015.681
8	6185.409
9	8260.823
10	5496.459
11	8662.853
12	6458.288
13	10437.723
14	6968.116
15	13649.815

Figure 4B, right

Figure 4C,D

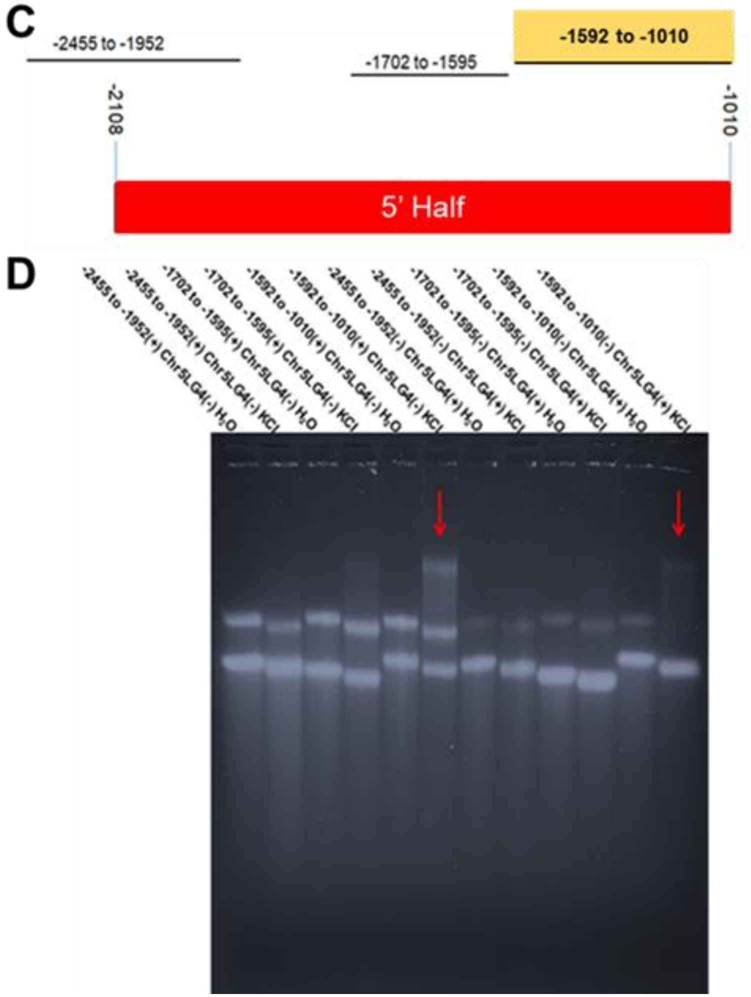
C
-2455 to -1952
-1702 to -1595
-1592 to -1010
-2108
-1010
5' Half
D
-2455 to -1952(+) Chr5LG4(-) H2O
-2455 to -1952(+) Chr5LG4(-) KCl
-1702 to -1595(+) Chr5LG4(-) H2O
-1702 to -1595(+) Chr5LG4(-) KCl
-1592 to -1010(+) Chr5LG4(-) H2O
-1592 to -1010(+) Chr5LG4(-) KCl
-2455 to -1952(-) Chr5LG4(+) H2O
-2455 to -1952(-) Chr5LG4(+) KCl
-1702 to -1595(-) Chr5LG4(+) H2O
-1702 to -1595(-) Chr5LG4(+) KCl
-1592 to -1010(-) Chr5LG4(+) H2O
-1592 to -1010(-) Chr5LG4(+) KCl

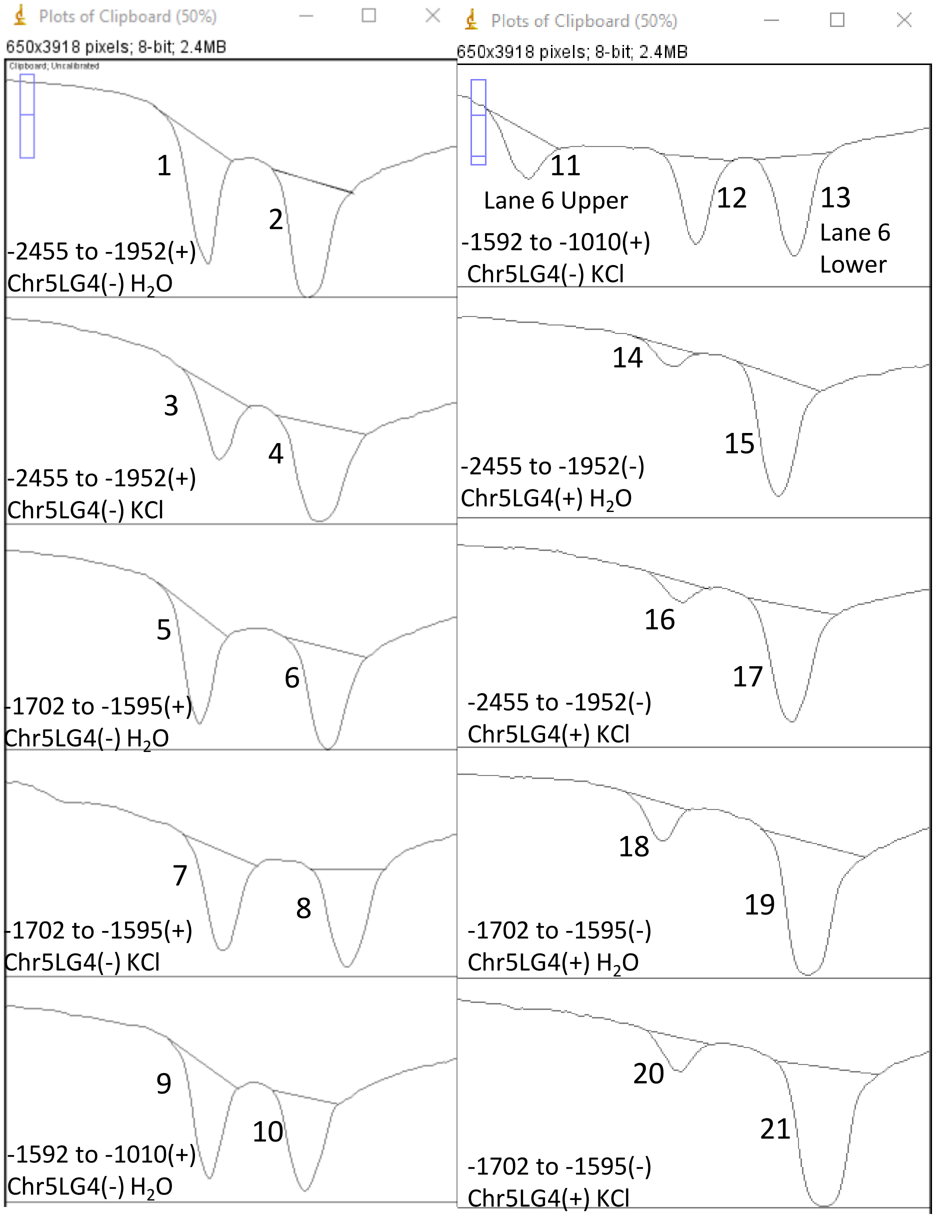

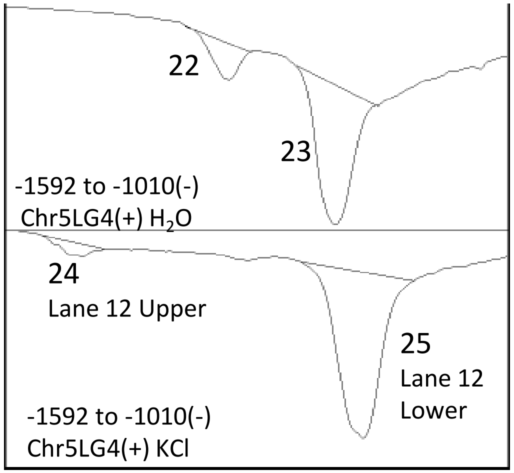

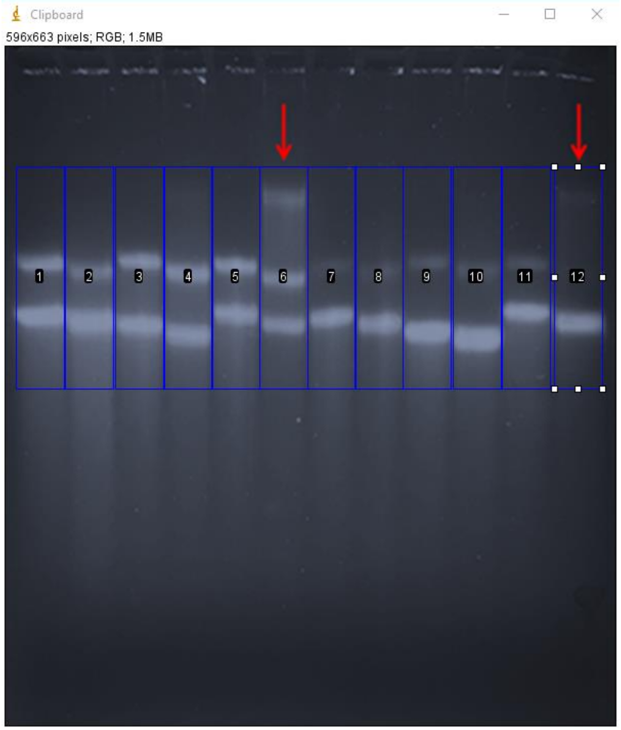

Results

File Edit Font

	Area
1	7756.844
2	10044.874
3	4683.116
4	9995.702
5	6959.208
6	8372.581
7	7431.702
8	7868.125
9	7069.430
10	6790.075
11	3490.409
12	5423.418
13	6919.075
14	1183.912
15	9134.652
16	1279.983
17	9537.288
18	2311.004
19	12819.966
20	2163.640
21	14473.116
22	1904.468
23	9664.066
24	818.598
25	14027.551

Figure 4E,F

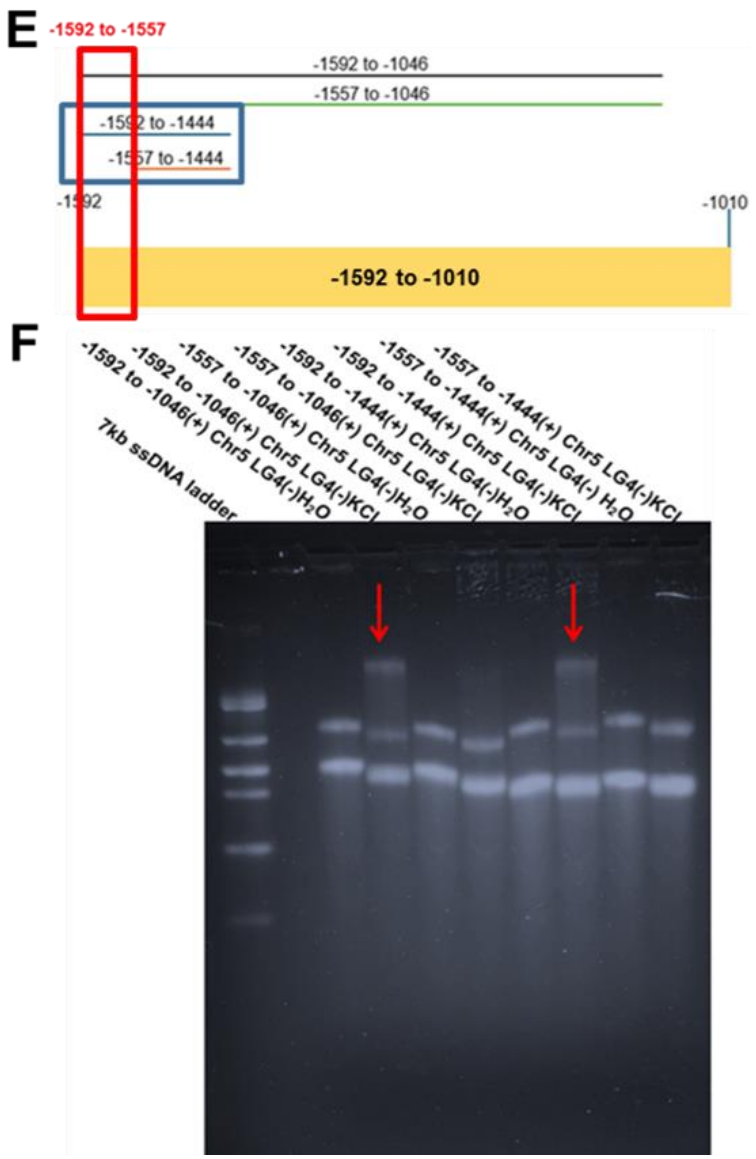
E
-1592 to -1557
-1592 to -1046
-1557 to -1046
-1592 to -1444
-1557 to -1444
-1592
-1010
-1592 to -1010
F
7kb ssDNA ladder
-1592 to -1046(+) Chr5 LG4(-)H_2O
-1592 to -1046(+) Chr5 LG4(-)KCl
-1557 to -1046(+) Chr5 LG4(-)H_2O
-1557 to -1046(+) Chr5 LG4(-)KCl
-1592 to -1444(+) Chr5 LG4(-)H_2O
-1592 to -1444(+) Chr5 LG4(-)KCl
-1557 to -1444(+) Chr5 LG4(-) H_2O
-1557 to -1444(+) Chr5 LG4(-)KCl

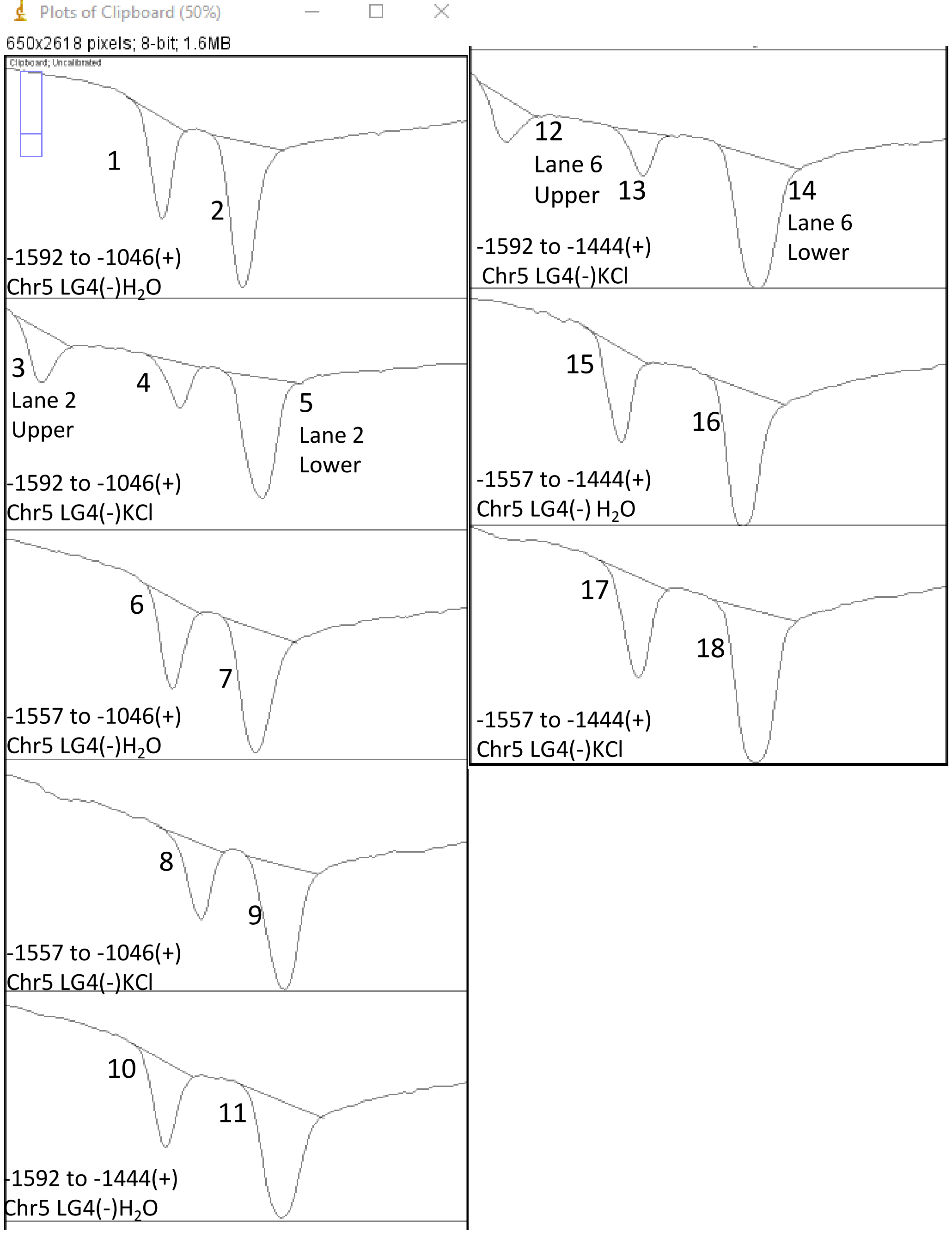

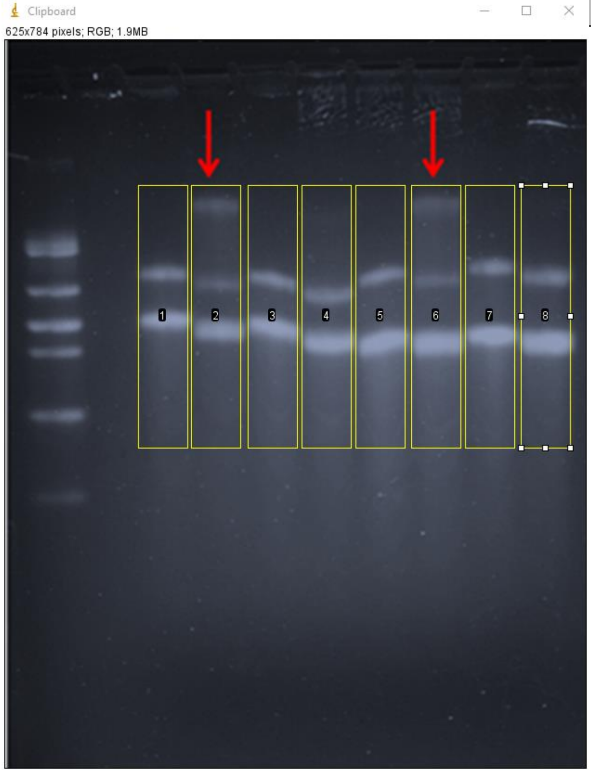

	Area
1	4705.489
2	8641.439
3	2922.024
4	2185.104
5	8424.489
6	4598.075
7	8576.924
8	4041.610
9	8827.853
10	4455.560
11	9366.066
12	2439.317
13	2042.154
14	10522.631
15	4574.782
16	9314.217
17	5602.388
18	11991.631

Figure 5B

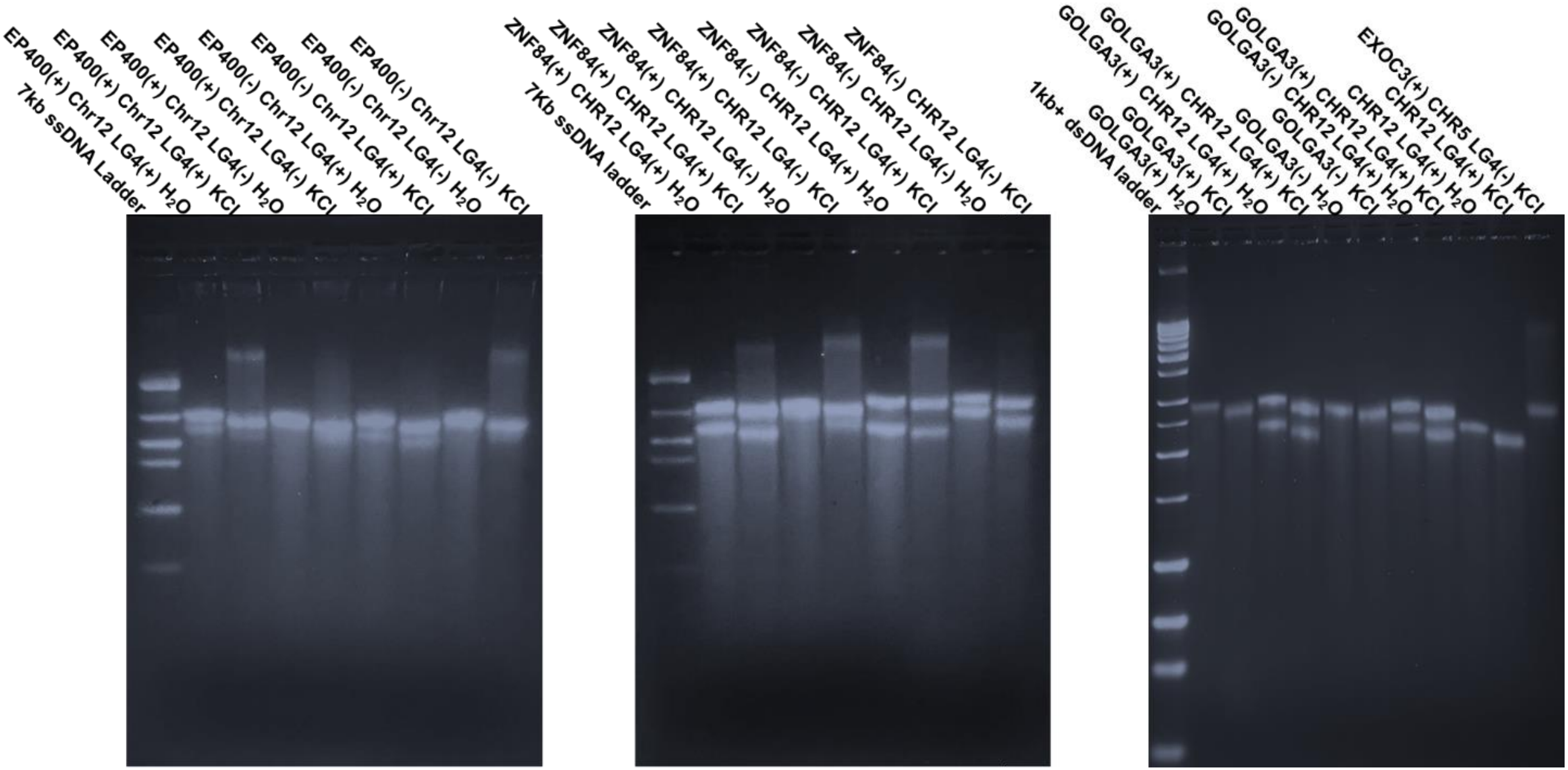
7kb ssDNA Ladder
EP400(+) Chr12 LG4(+) H2O
EP400(+) Chr12 LG4(+) KCl
EP400(+) Chr12 LG4(-) H2O
EP400(+) Chr12 LG4(-) KCl
EP400(-) Chr12 LG4(+) H2O
EP400(-) Chr12 LG4(+) KCl
EP400(-) Chr12 LG4(-) H2O
EP400(-) Chr12 LG4(-) KCl
7Kb ssDNA ladder
ZNF84(+) CHR12 LG4(+) H2O
ZNF84(+) CHR12 LG4(+) KCl
ZNF84(+) CHR12 LG4(-) H2O
ZNF84(+) CHR12 LG4(-) KCl
ZNF84(-) CHR12 LG4(+) H2O
ZNF84(-) CHR12 LG4(+) KCl
ZNF84(-) CHR12 LG4(-) H2O
ZNF84(-) CHR12 LG4(-) KCl
1kb+ dsDNA ladder
GOLGA3(+) H2O
GOLGA3(+) KCl
GOLGA3(+) CHR12 LG4(+) H2O
GOLGA3(+) CHR12 LG4(+) KCl
GOLGA3(-) H2O
GOLGA3(-) KCl
GOLGA3(-) CHR12 LG4(+) H2O
GOLGA3(+) CHR12 LG4(+) KCl
CHR12 LG4(+) H2O
CHR12 LG4(+) KCl
EXOC3(+) CHR5 LG4(-) KCl

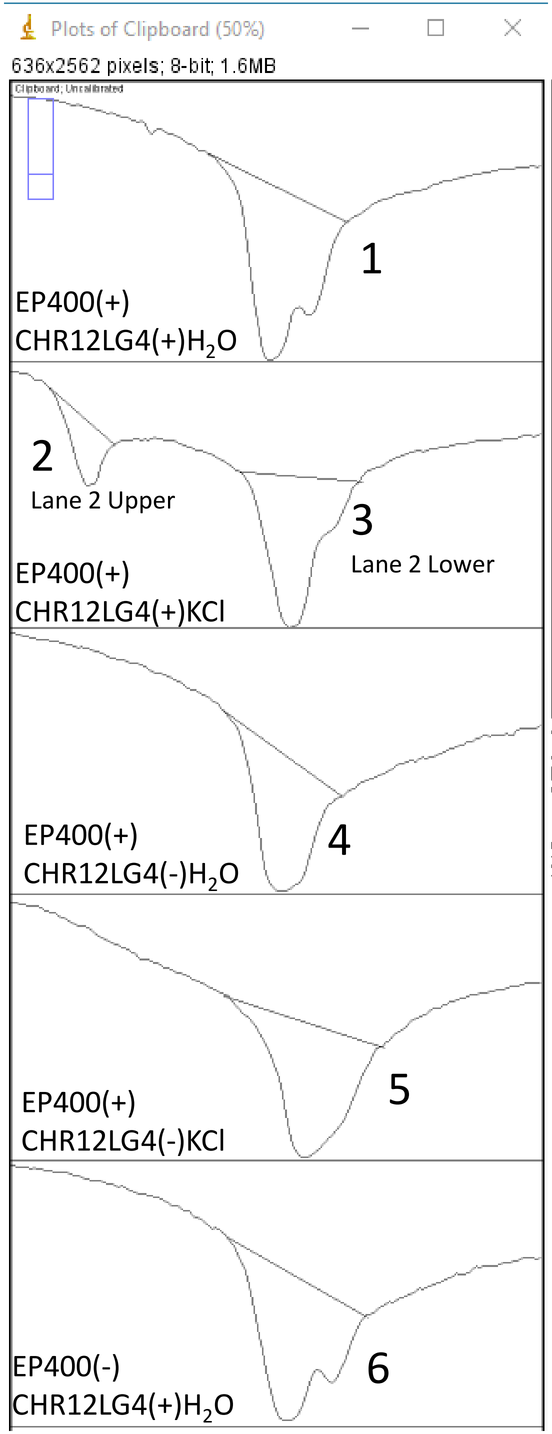

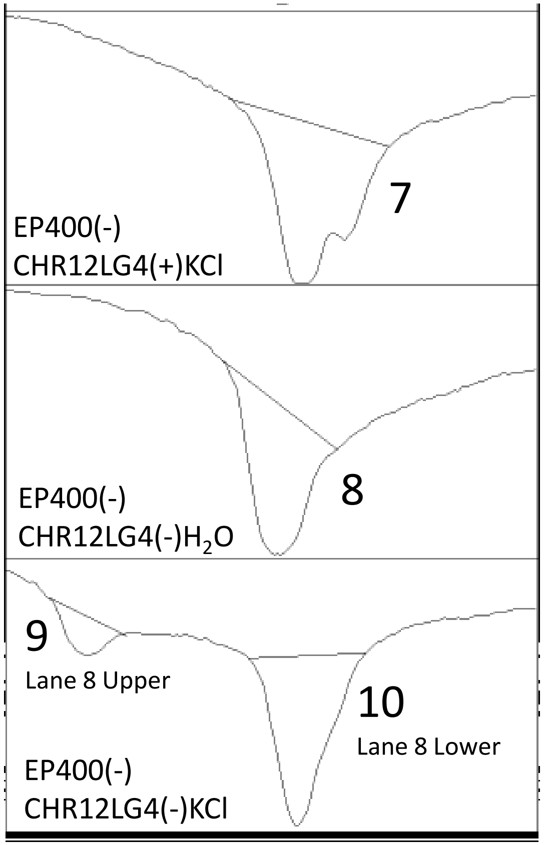

File Edit Font

	Area
1	16189.978
2	2976.631
3	12103.823
4	11863.735
5	14249.978
6	15390.291
7	17734.735
8	12859.978
9	2362.418
10	13079.530

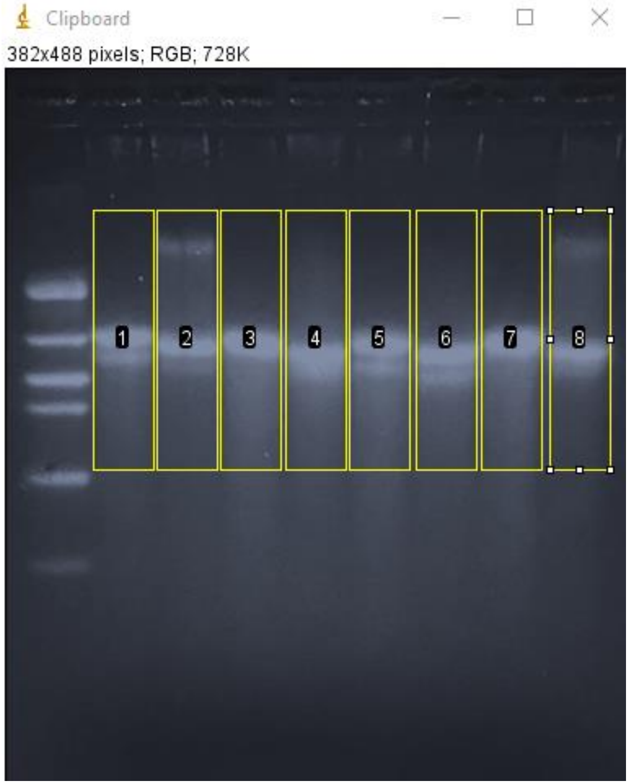

Figure 5B left

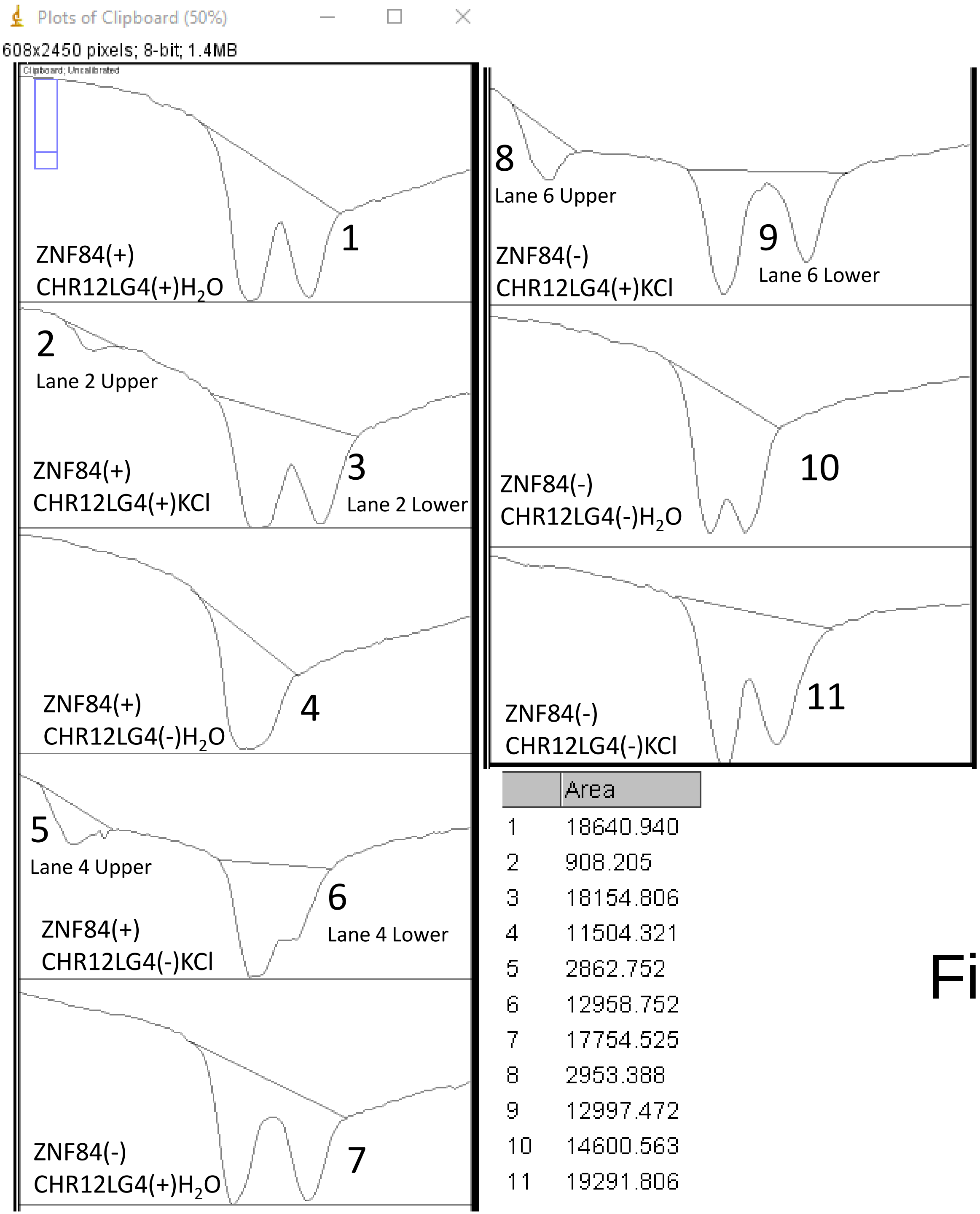

	Area
1	18640.940
2	908.205
3	18154.806
4	11504.321
5	2862.752
6	12958.752
7	17754.525
8	2953.388
9	12997.472
10	14600.563
11	19291.806

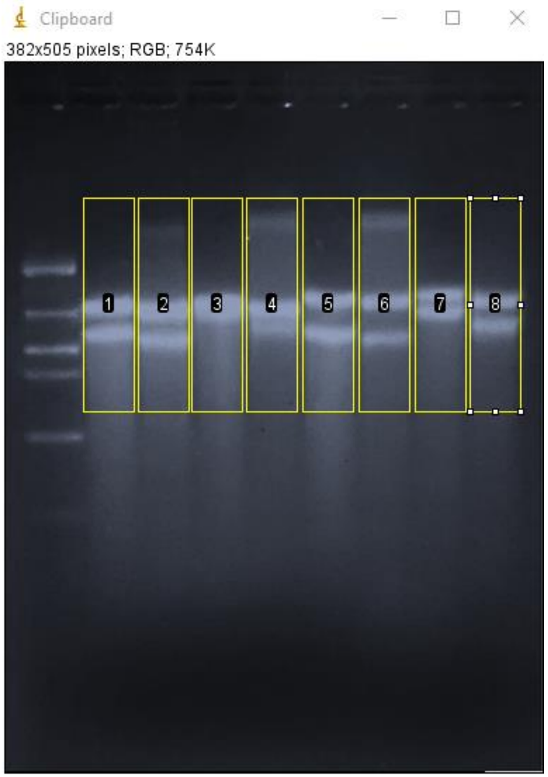

Figure 5B middle

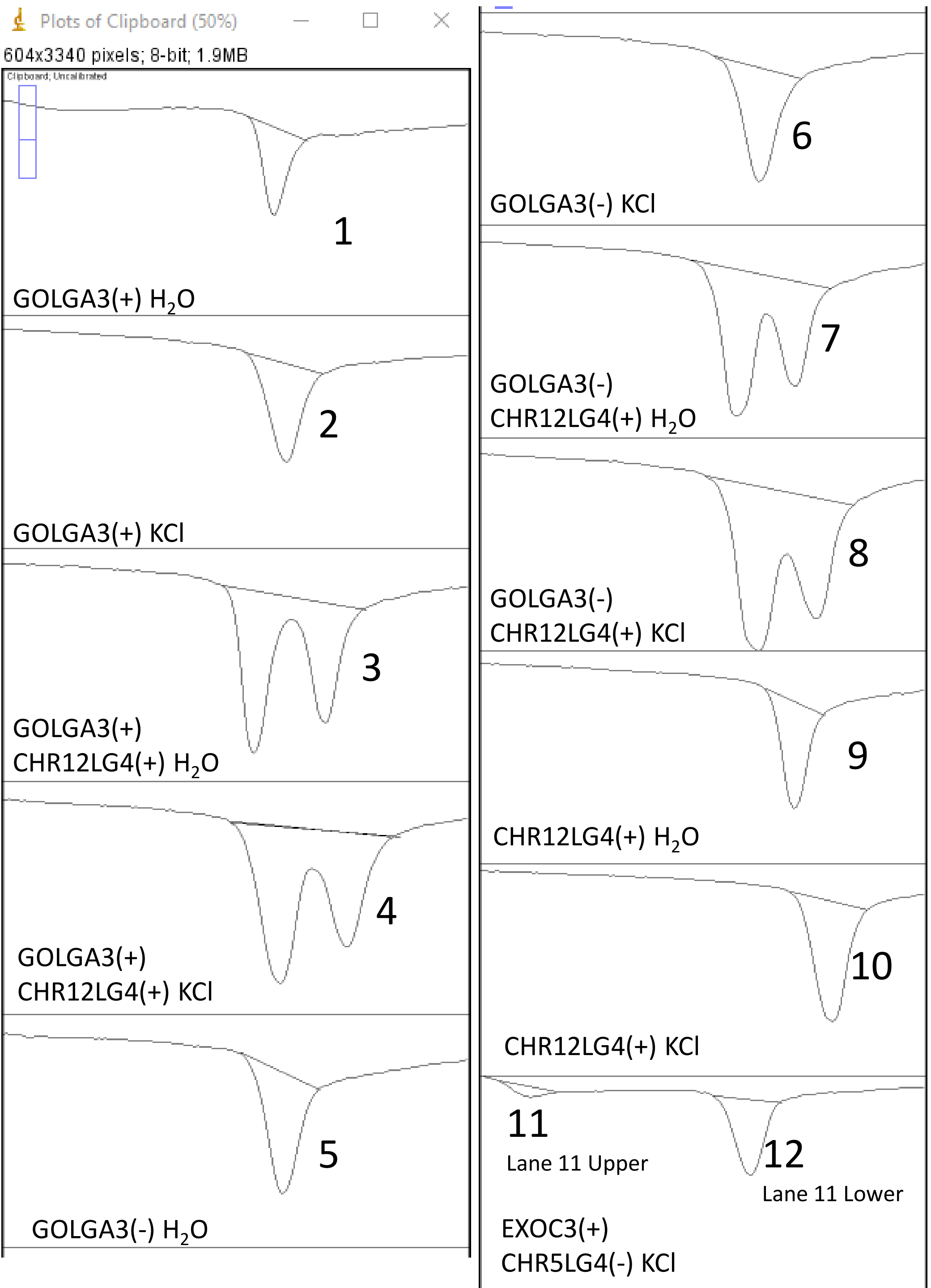

	Area
1	3867.418
2	5884.974
3	15523.836
4	19101.028
5	7115.823
6	8125.409
7	17428.078
8	22901.392
9	5709.024
10	8442.388
11	696.820
12	4628.761

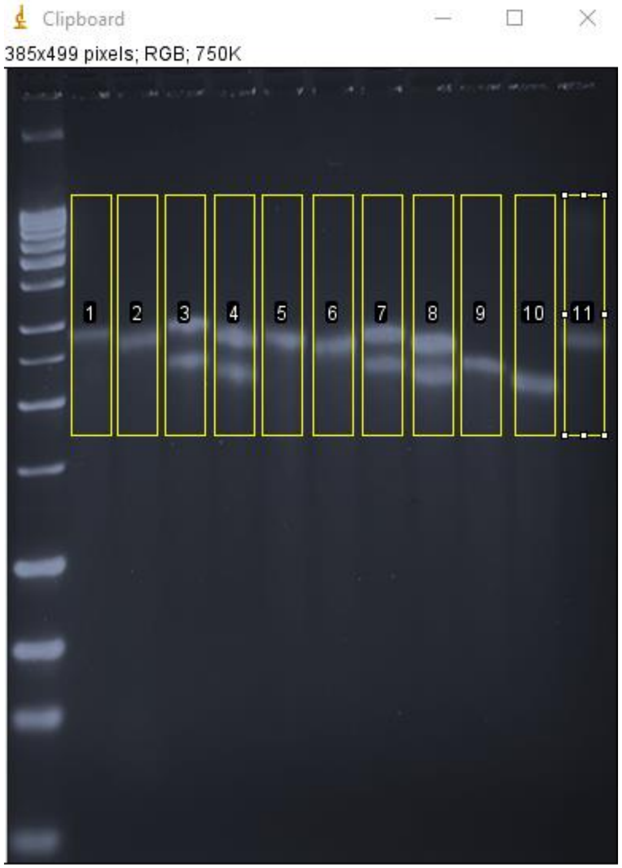

Figure 5B right

Exoc3prom 5’half (-2108 to -1127) *overlap with Middle 300bp has been removed*

CACGTCCTTGCCTGTTCGTGAATCCACTTGTGCAATCGACATGATGCTTGCACTCAGCAGTGCTCCTGGGTGTTCGCAGAACATGGTCTGCCAAAAACAGAAGTCAAGAAAC
TTTTTTTTTGAGACAGATTCTCCCTCTGTCACCCAGGCTGGAGTGTAGTGGTGCCATCTCAGTTCACTGCAACCTCCTCCTCCCGGGTTGAAGCCATTCTCCTGCCTCAGC
CTCCCAGGGAGCTGAGATTACAGGCATGCACCACCATGCCTGGCTAATTTTTGTGGGGTTTTTTTTGGTAGAGATGGGGTTCCTACCAAAAAGACCATGTTGCCCAGGCTGG
TCTCAAACTCCTGGGCTCAAGGGGTCTGCCCATCTCAGCCTCCCAAAGTGCTGGGATTACAGTTAGGAGCCACTGCGCCCAGCCAATAAATACCTTTTTTTTTTTTTTTTT
TTTTTTTTTTTTTTGAGACGGAGCCTCGCTCTGTTGCCCAGGCTGGAGTACAGTGGCGCGATCTTGGCTCACTGCAAGCTCCGCCTCCCGGGTTCACGCCATTCTCCTGCCT
CAGCCTCCCGAGTAGCTGGGACTCCAGGGGCCCACCACCACACCTGCCTAGTTTTTTTGTATTTTTTTTAGTAGAGACGGGGTTTCACCATGTTAGCCAGGATGGTCTCGAT
CTCCTGACCTCATGATCTACCTGCCTCGCCTCCCAAAGTGCTGGGATTACAGGCGTGAGCCACGGCGCCCCGCCAAGAAAGACTTCTAATCAATGTTTTGTTAAGAAGTTAC
TAGACAGTCCTCCATCCAAATTCACTACCAGACACTGATATAATAGTATGAGTCTTTAGTTCATAATACTCAATGTTACTATATTTTTAAGAGTCCTAAAAGCTTTGGCAAT
TTTGGCAATTTTTTCTTCATTAGTTTCAAAATTTCTTTACAGTCATAATTTCTAACACAATTTTAACTTTTTTACTTTTTTTCATT

Exoc3prom Middle 300bp (-1126 to -828)

GCTCAACTTGTTGCTAGATTTTCAGAAATTACAAGGAAAAACTGAAATAATAGAGCCGGGGTCAGTGTCAACATCAGCAGGGCACGGGGCAGGGCTCCCTGTCGGAGTGGCA
GTGGGGGCTCCCGCAAGCTCCATCTCCTTCCTGTGTGCCCCTGCCCCATGCTGCTGCCACTTCCCTGGTCTCTGCTGGCTTCCCTGCCACCTTCCAGACCCTTCCTTTCGTG
TGGCTAATTTTCAGGAGGGGCAGGGGCACAACCTAGCAGCTCCAACCTATGAAAGACTTTGTCAGTTGGTGTGTC

Exoc3prom 3’half (-827 to +136) TSS = Green *overlap with Middle 300bp has been removed*

AAAGCCAGCATCTTTTATCAAACAGACAAACCCTGAACGCTCGTGGTTTAGCACGTGCTTCTTCCTCCAGGCGAAGCCAAGTGCTGGCCATGGGCCTCTCCTCCAGGAAGCG
ACTCAGGACCGGCCCTCTGCAGCGCGGCCCAGTGACCCACATTCATTCTCCAAGACCTGCGCTGCTGGGCCTCTGAGGGGCCCCGGAAACATCCGGTGGCCACCCATCCCAC
TGGCATTGTTCCCACGAGGGTGCCAATGACTGCGCTCCTGGAGGGGCTGAGTTCGTCTTTGGGTCCAGGAGGTACCCAAAGCCGCTTGTCTGCTGGTGGCAGGTATGACAGA
CGTTTGATTCCCAACCTTCTCCGCTTTGGTTTCTTCGTTTACTGAATGCCCGGCGCCCACCTCAGTCCGCGGGAAACGAGGGACGCCCCTGCCACACTGCAGTGCGGACGAC
GAGGCCAACATAAAGACAGCAGGCATCAGCAGAGGATGGAGACAGCGGCTCTGGGGCTGTCTTCTCTCCGCAGGTTGGGAAGGGAAAGGAGAGAAAATGCACGCAGTCACGC
TCGGCGGAGGCGTCCAGGACTGCGCCCGCCCTGGGGAAGGGCGCACGCGGTGGTCGCCAGGGGCAGCGAGAGGGCCTGAGTTGGGGGGCTGGGACGGACAAGAAGCCCCCC
ACCTCGGCGGTTTCCCTGTCCTGCTGGGGAGCGGCCATGGGGCAGGTCCCCGAGCTGAAGTCGCTCGGCCGGGAGACGAGTACCCGCCCCGCGGGAGGGCCCGGACCCGTGC
TTCCGGCGCCGTTGCCGGAAGCGGAAGTGCCCGCGCGCTGTCCACTTCCGGCCGGGACCCCGGAGGCGGAGGCAGCGAAGGCGGAGGGGGCGGCGGGGGCGGCGGCGGCGGC
GGCGGCGGCGGCGGCGGCGGCGGCGGCGGCGGCGTAGCCGTAGAGGGTGAGTCGGTGGCAGGTCCTG

-1592 to -1046
TCACTGCAAGCTCCGCCTCCCGGGTTCACGCCATTCTCCTGCCTCAGCCTCCCGAGTAGCTGGGACTCCAGGGGCCC
ACCACCACACCTGCCTAGTTTTTTTGTATTTTTTTTAGTAGAGACGGGGTTTCACCATGTTAGCCAGGACGGTCTCG
ATCTCCTGACCTCATGATCTACCTGCCTCGCCTCCCAAAGTGCTGGGATTACAGGCGTGAGCCACGGCGCCCCGCCA
AGAAAGACTTCTAATCAATGTTTTGTTAAGAAGTTACTAGACAGTCCTCCACCCAAATTCACTACCAGACACTGATA
TAATAGTATGAGTCTTTAGTTCATAATACTCAATGTTACTGTATTTTTAAGAGTCCTAAAAGCTTTGGCAATTTTGG
CAATTTTTTCTTCATTAGTTTCAAAATTTCTTTACAGTCATAATTTCTAACACAATTTTAACTTTTTTACTTTTTTT
CATTGCTCAACTTGTTGCTAGATTTTCAGAGATTACAAGGAAAAACTGAAATAATAGAGCCGGGGTCAGTGTCAACA
TCAGCAGG

-1557 to -1046
TTCTCCTGCCTCAGCCTCCCGAGTAGCTGGGACTCCAGGGGCCCACCACCACACCTGCCTAGTTTTTTTGTATTTTT
TTTAGTAGAGACGGGGTTTCACCATGTTAGCCAGGACGGTCTCGATCTCCTGACCTCATGATCTACCTGCCTCGCCT
CCCAAAGTGCTGGGATTACAGGCGTGAGCCACGGCGCCCCGCCAAGAAAGACTTCTAATCAATGTTTTGTTAAGAAG
TTACTAGACAGTCCTCCACCCAAATTCACTACCAGACACTGATATAATAGTATGAGTCTTTAGTTCATAATACTCAA
TGTTACTGTATTTTTAAGAGTCCTAAAAGCTTTGGCAATTTTGGCAATTTTTTCTTCATTAGTTTCAAAATTTCTTT
ACAGTCATAATTTCTAACACAATTTTAACTTTTTTACTTTTTTTCATTGCTCAACTTGTTGCTAGATTTTCAGAGAT
TACAAGGAAAAACTGAAATAATAGAGCCGGGGTCAGTGTCAACATCAGCAGG

-1557 to -1444
TTCTCCTGCCTCAGCCTCCCGAGTAGCTGGGACTCCAGGGGCCCACCACCACACCTGCCTAGTTTTTTTGTATTTTT
TTTAGTAGAGACGGGGTTTCACCATGTTAGCCAGGA

-1592 to -1444
TCACTGCAAGCTCCGCCTCCCGGGTTCACGCCATTCTCCTGCCTCAGCCTCCCGAGTAGCTGGGACTCCAGGGGCCC
ACCACCACACCTGCCTAGTTTTTTTGTATTTTTTTTAGTAGAGACGGGGTTTCACCATGTTAGCCAGGATGG

-2455 to -1952
CCCTCGAGTTTGCTCCTGTCAAGGTGGAGGAGGATCCCTCTAGCAATGACCCTGGGTTGGCTTCCGCCGAGGGCTGA
GTTTAGGAACAGTCTCCAGGCTGTTGCTGTTTTCCTAGTCCACGCTTTCACTGAGGATGCATCCCTTGCACATCTGT
GCTTCACATGGGAGTCGCCGTCCACCTCCCACCTGCTGGGAGCCTTAGCTTCATCATCTGTCCTGCTTCAGTGTAAA
ACCCAACCCCTGGGCCGCAGAACTCGCACCCTACCCACCCACCTGCCTGCTGTAGGACAGCAAGCATCTCTGCTTCT
GATTCACGTCCTTTCCCCTTCCCGCATTTGTCCCTGGTCACGTCCTCGCCTGTTCGTGAATCCACTTGTGCAATCGA
CATGATGCTTGCACTCAGCAGTGCTCCTGGGTGTTCGCAGAACATGGTCTGCCAAAAACAGAAGTCAAGAAACTTTT
TTTTTTGAGACAGATTCTCCCTCTGTCACCCAGGCTGGAGTACAGTGGCGCGATCT

-1592 to -1010
TCACTGCAAGCTCCGCCTCCCGGGTTCACGCCATTCTCCTGCCTCAGCCTCCCGAGTAGCTGGGACTCCAGGGGCCC
ACCACCACACCTGCCTAGTTTTTTTGTATTTTTTTTAGTAGAGACGGGGTTTCACCATGTTAGCCAGGACGGTCTCG
ATCTCCTGACCTCATGATCTACCTGCCTCGCCTCCCAAAGTGCTGGGATTACAGGCGTGAGCCACGGCGCCCCGCCA
AGAAAGACTTCTAATCAATGTTTTGTTAAGAAGTTACTAGACAGTCCTCCACCCAAATTCACTACCAGACACTGATA
TAATAGTATGAGTCTTTAGTTCATAATACTCAATGTTACTGTATTTTTAAGAGTCCTAAAAGCTTTGGCAATTTTGG
CAATTTTTTCTTCATTAGTTTCAAAATTTCTTTACAGTCATAATTTCTAACACAATTTTAACTTTTTTACTTTTTTT
CATTGCTCAACTTGTTGCTAGATTTTCAGAGATTACAAGGAAAAACTGAAATAATAGAGCCGGGGTCAGTGTCAACA
TCAGCAGGGCACGGGGCAGGGCTCCCTGTCGGAGTGGCAGTGGG

-1702 to -1595
CACTGTGCCCAGCCAATAAATCCCTTTTTTTTTTTTTTTTTTTTTTTTTTTTTTTTTTTGAGACGGA
GCCTCGCTCTGTTGCCCAGGCTGGAGTACAGTGGCGCGATTCTTG

Supplemental Figure 4. EXOC3 promoter deletion construct sequences. Construct names correspond to the regions indicated in **Figure 4**. Numbers preceding sequences refer to nt position with respect to the primary EXOC3 TSS (highlighted in Green in Exoc3prom 3’ half). Regions in the 5’ half and 3’ half constructs overlapping the Middle 300bp construct are only included in the Middle 300bp construct. ≥3 consecutive genomic Gs shown in red and ≥3 consecutive genomic Cs shown in blue.

In-trans luciferase assay

In-cis luciferase assay

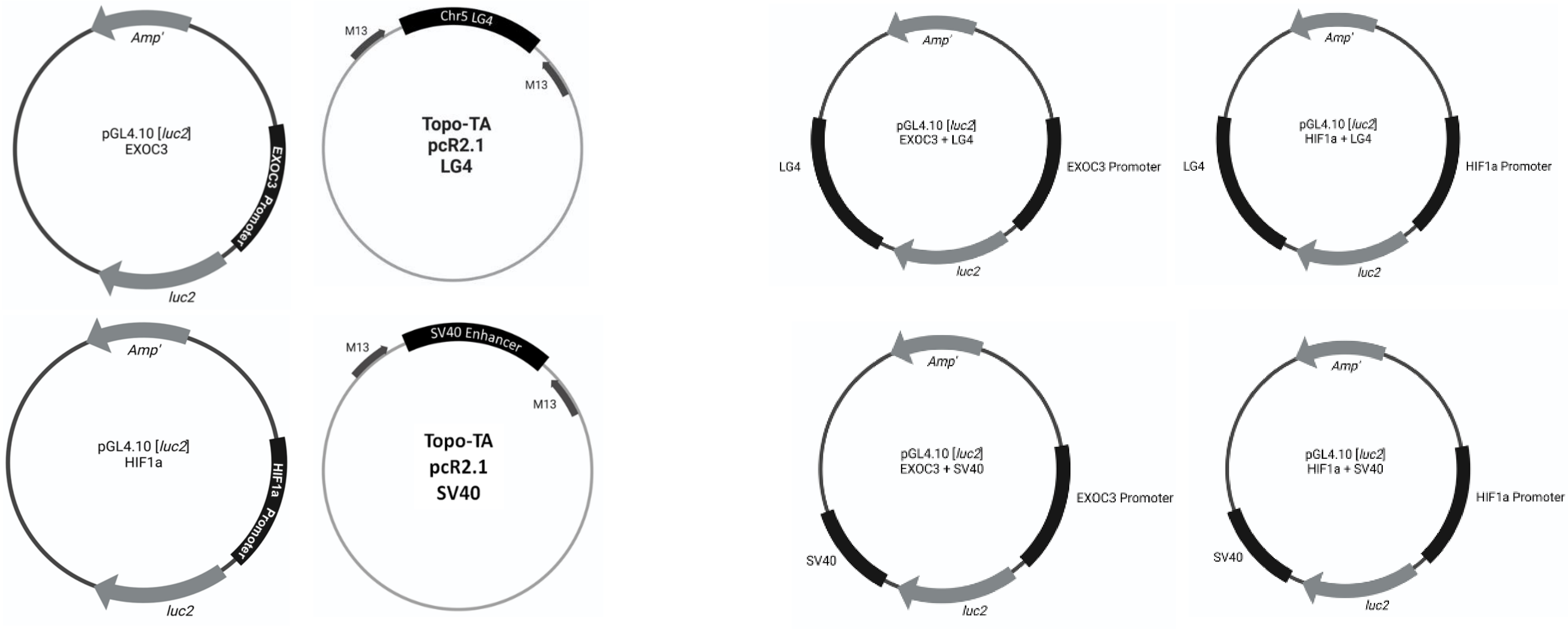

Supplemental Figure 5. Luciferase assay constructs. In-trans luciferase assays utilize a reporter plasmid pGL4.10 [luc2] (left) under transcriptional control of a specified promoter element EXOC3 (top) or HIF1a (bottom) and also a separate plasmid, Topo-TA pcR2.1 (right) containing a specified enhancer element such as the Chr5 LG4 (top) or SV40 universal enhancer (bottom). In-cis luciferase assays utilize reporter plasmids containing a promoter, EXOC3 (left) or HIF1a (right) and also an enhancer element Chr5 LG4 (top) or SV40 universal enhancer (bottom).

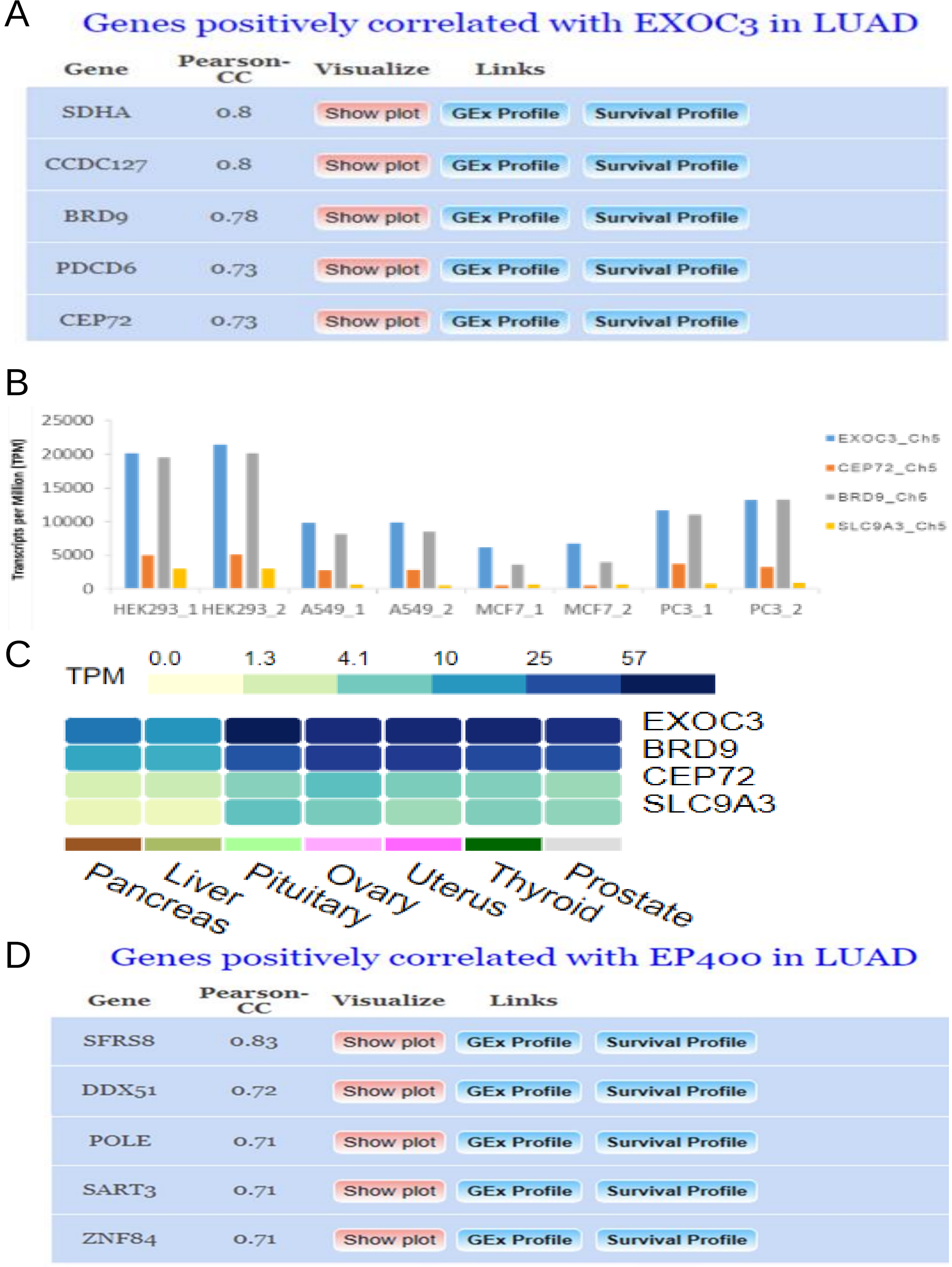

Supplemental Figure 6. Expressions of genes regulated by LG4 enhancers are coregulated. (A) Screen capture of UALCAN entry[SF2(1),SF2(2)] for the top 5 genes positively correlated with EXOC3 in lung adenocarcinoma (LUAD) TCGA paient samples. **(B)** Chr5 LG4 target gene expressions in available NCBI GEO datasets taken from two distinct isolates of human lung (A549), breast (MCF7), prostate (PC3) and kidney (HEK293) cell lines as determined by standard Borchert Lab protocols [SF2(3)]. **(C)** Screen capture of GTEx Multi Gene Query [SF2(4)]. **(D)** Screen capture of UALCAN entry for top genes positively correlated with EP400 in lung adenocarcinoma (LUAD) TCGA patient samples.

SF2(1) Chandrashekar DS, Karthikeyan SK, Korla PK, Patel H, Shovon AR, Athar M, Netto GJ, Qin ZS, Kumar S, Manne U, Creighton CJ, Varambally S. UALCAN: An update to the integrated cancer data analysis platform. Neoplasia. 2022 Mar;25:18-27. doi: 10.1016/j.neo.2022.01.001 [PMID: 35078134]

SF2(2) Chandrashekar DS, Bashel B, Balasubramanya SAH, Creighton CJ, Rodriguez IP, Chakravarthi BVSK and Varambally S. UALCAN: A portal for facilitating tumor subgroup gene expression and survival analyses. Neoplasia. 2017 Aug;19(8):649-658. doi: 10.1016/j.neo.2017.05.002 [PMID:28732212]

SF2(3) Williams, J. D. et al. Characterization of long G4-rich enhancer-associated genomic regions engaging in a novel loop:loop ‘G4 Kissing’ interaction. Nucleic Acids Res. 48, 5907–5925 (2020)

SF2(4) Consortium GT, Laboratory DA, Coordinating Center - Analysis Working G, Statistical Methods groups-Analysis Working G, Enhancing Gg, Fund NIHC, et al. Genetic effects on gene expression across human tissues. Nature. 2017; 550(7675):204–13. https://doi.org/10.1038/nature24277 PMID: 29022597

Supplemental Table 1. Select Pore-C read genomic alignments.

Read SRR11589412.3086865.1

Genomic Location	Overlapping Gene	Overlapping Regulatory Feature	Strand	Start (nt)	Stop (nt)	Length	% ID
22:29293539-29293772	EWSR1		Forward	8	240	234	98.7
22:29345837-29346494	AP1B1		Forward	235	895	669	96.4
22:27089457-27089985			Reverse	901	1432	539	96.3
22:27717382-27717674	ENSG00000226169		Forward	1429	1724	297	97.6
22:27636691-27637040			Reverse	1721	2086	371	92.7
22:27737965-27738414			Forward	2087	2535	454	95.6
**5:427872-428040**	PDCD6-AHRR, AHRR	PDCD6-AHRR Internal Enhancer	Forward	2530	2700	171	98.8
**5:550713-550930**			Reverse	2694	2916	226	94.2
**5:555318-555631**	**LG4**	**5:551937-556935**	Forward	2916	3222	320	92.8
7:154800300-154800638	DPP6		Reverse	3226	3561	339	97.6
22:31571804-31572080	SFI1		Reverse	3555	3823	278	96.0
22:31190803-31190885	RNF185		Forward	3818	3899	83	98.8
22:30477788-30478402	ENSG00000181123		Reverse	3902	4506	621	94.7
**5:441750-442211**	**EXOC3-AS1**	**EXOC3 Promoter**	Forward	4507	4970	468	96.2
8:107935200-107935634	ENSG00000287949, RSPO2		Forward	4971	5398	441	94.3
**5:463134-463368**	EXOC3	EXOC3 Internal Enhancer	Reverse	5393	5626	237	95.8
**5:583782-585405**	2nd LG4	5:583490-585428	Forward	5622	7269	1647	97.2
22:29241083-29241553	EMID1		Reverse	7354	7820	475	96.2
**5:462232-462368**	EXOC3	EXOC3 Internal Enhancer	Forward	7817	7954	138	96.4
**5:462369-462924**	EXOC3	EXOC3 Internal Enhancer	Forward	7955	8512	566	94.9
**5:695341-695648**		TPPP Promoter	Reverse	8509	8820	313	98.1
**5:679888-680532**	TPPP	TPPP Variant Promoter	Forward	8817	9463	667	91.6
8:106523244-106523440	OXR1-AS1, OXR1		Forward	9460	9658	200	98.0
22:31796071-31796341	DEPDC5, ENSG00000285404		Reverse	9655	9926	274	97.8
22:31798546-31798674	DEPDC5, ENSG00000285404		Reverse	9927	10052	125	96.2
22:31954084-31954738	YWHAH, ENSG00000285404		Reverse	10053	10690	669	91.6
**5:523922-525775**	SLC9A3, ENSG00000250385	SLC9A3 Promoter	Reverse	10701	12540	1914	89.7
**5:420780-422473**	PDCD6-AHRR, AHRR	PDCD6-AHRR Internal Enhancer	Forward	12560	14248	1732	93.8
						Avg. % ID	95.7

Read SRR11589401.9930809.1

Genomic Location	Overlapping Gene(s)	Overlapping Regulatory Feature	Strand	Start (nt)	Stop (nt)	Length	%ID
8:143720044-143720646	MAPK15		Forward	255	841	611	92.80
3:47207529-47207677	KIF9-AS1		Reverse	838	982	150	94.67
12:132196795-132197858	GALNT9		Forward	979	2024	1082	93.35
10:2185537-2186447	LINC02662		Reverse	2021	2909	917	95.20
4:35003947-35004937			Reverse	2906	3899	1007	95.93
12:132652603-132652816	POLE		Reverse	3896	4107	217	94.47
5:167466545-167466965	TENM2		Forward	4104	4522	429	93.24
**12:133037754-133038269**	**ZNF84**	**ZNF84 Promoter**	Reverse	4516	5024	523	92.16
12:132257769-132258365	GALNT9		Forward	5022	5604	608	90.79
**12:132698890-132699138**	**PXMP2, ENSG00000256632**	**Enhancer**	Forward	5600	5840	250	95.60
8:144786494-144788109	ZNF34	ZNF34 Promoter, CTCF	Forward	5837	7402	1639	92.01
12:132740734-132741801	ANKLE2		Reverse	7398	8435	1081	92.41
12:132751579-132751706	ANKLE2		Reverse	8426	8553	129	96.12
						Avg. % ID	93.75

Supplemental Table 4. Select Pore-C reads containing segments of both an LG4 and its putative target promoter.

Reads containing sequences from both the Chr5 LG4 and the EXOC3 promoter

Read ID	LG4 Genomic Alignment(s)	SRA Study ID	Sample Type
gnl|SRA|SRR11589410.583278.1	5:548110-548860	SRX8156772	Breast Cancer
gnl|SRA|SRR11589413.15692494.1	5:551435-551730	SRX8156772	Breast Cancer
gnl|SRA|SRR11589412.3086865.1	5:550710-550930, 5:555318-555631	SRX8156772	Breast Cancer
gnl|SRA|SRR11589410.673842.1	5:549956-550672	SRX8156772	Breast Cancer
gnl|SRA|SRR18173259.11055807.1	5:551086-551339	SRX14320238	Prostate Cancer
gnl|SRA|SRR18173258.9524917.1	5:551082-551340	SRX14320238	Prostate Cancer
gnl|SRA|SRR18173259.12581060.1	5:558131-558485	SRX14320238	Prostate Cancer
gnl|SRA|SRR18173261.2238679.1	5:551725-553887	SRX14320239	Prostate Cancer
gnl|SRA|SRR18173260.15552004.1	5:550629-550930	SRX14320240	Prostate Cancer
gnl|SRA|SRR18173261.16302190.1	5:550156-550630	SRX14320241	Prostate Cancer
gnl|SRA|SRR18173260.4738883.1	5:554702-554976	SRX14320242	Prostate Cancer
gnl|SRA|SRR18173260.14713866.1	5:554508-554977	SRX14320243	Prostate Cancer

Supplemental Table 5. LG4 regulatory neighborhood gene fusions.

Chromosome 5 Gene Fusion Events

FusionGID	FusionGene	Hgene	HGID	Tgene	TGID
15891	CEP72-EXOC3	CEP72	55722	EXOC3	11336
10223	BRD9-CEP72	BRD9	65980	CEP72	55722
83551	SLC9A3-ZDHHC11	SLC9A3	6550	ZDHHC11	79844
83552	SLC9A3-ZDHHC11B	SLC9A3	6550	ZDHHC11B	653082
27895	EXOC3-TRIP13	EXOC3	11336	TRIP13	9319
10230	BRD9-TPPP	BRD9	65980	TPPP	11076
93435	TPPP-BRD9	TPPP	11076	BRD9	65980
15889	CEP72-AHRR	CEP72	55722	AHRR	57491
66270	PLEKHG4B-AHRR	PLEKHG4B	153478	AHRR	57491
3141	AHRR-PLEKHG4B	AHRR	57491	PLEKHG4B	153478
63622	PDCD6-SDHA	PDCD6	10016	SDHA	6389
66274	PLEKHG4B-PDCD6	PLEKHG4B	153478	PDCD6	10016
13609	CCDC127-SDHA	CCDC127	133957	SDHA	6389

Chromosome 12 Gene Fusion Events

FusionGID	FusionGene	Hgene	HGID	Tgene	TGID
4502	ANKLE2-POLE	ANKLE2	23141	POLE	5426
33879	GOLGA3-POLE	GOLGA3	2802	POLE	5426
66933	POLE-GOLGA3	POLE	5426	GOLGA3	2802
70903	PXMP2-GOLGA3	PXMP2	5827	GOLGA3	2802
33872	GOLGA3-CHFR	GOLGA3	2802	CHFR	55743
33887	GOLGA3-ZNF891	GOLGA3	2802	ZNF891	101060200
4497	ANKLE2-CHFR	ANKLE2	23141	CHFR	55743
26766	EP400-DDX51	EP400	57634	DDX51	317781
29584	FBRSL1-GALNT9	FBRSL1	57666	GALNT9	50614
4501	ANKLE2-NOC4L	ANKLE2	23141	NOC4L	79050
4505	ANKLE2-ZNF605	ANKLE2	23141	ZNF605	100289635
101495	ZNF140-ZNF268	ZNF140	7699	ZNF268	10795
16434	CHFR-ZNF605	CHFR	55743	ZNF605	100289635
29588	FBRSL1-NOC4L	FBRSL1	57666	NOC4L	79050
